# Inferential procedures for random effects in generalized linear mixed models

**DOI:** 10.1371/journal.pone.0320797

**Published:** 2025-04-16

**Authors:** Xu Ning, Francis K.C. Hui, Alan Welsh

**Affiliations:** Research School of Finance, Actuarial Studies and Statistics, The Australian National University, Canberra, ACT, Australia; Indian Statistical Institute, INDIA

## Abstract

We study three commonly applied measures of uncertainty for random effects prediction in generalized linear mixed models (GLMMs), namely the unconditional and conditional mean squared errors of prediction (UMSEP and CMSEP, respectively), and the unconditional variance of the prediction gap used by the popular R package for glmmTMB. We demonstrate that, although the three theoretical measures differ in how they quantify uncertainty, the resulting estimators all turn out to be very similar in form. We derive asymptotic results regarding the consistency of the three measures of uncertainty, and in doing so resolve a contradiction between theoretical and empirical results for the glmmTMB variance estimator by re-interpreting it conditionally on a finite subset of the random effects. Our results have important implications for predictive inference in GLMMs, particularly around the legitimacy and implications of coupling these measures with a normality assumption to construct prediction intervals for the random effects.

## 1 Introduction

Generalized linear mixed models (GLMMs) are widely used to analyze clustered data e.g., in longitudinal and multilevel studies [1–3]. In GLMMs, unobserved random effects included in the linear predictor induces correlations between observations within a cluster; these random effects play an important role in many applications e.g., predictions in small-area estimation [4,5], and their realized values or functions thereof are often of direct interest to domain experts [5–7]. The empirical distribution of the predicted random effects is also often examined in model diagnostics, e.g., [8–10], to assess whether the normality assumption for the random effects is reasonable.

For the empirical Bayes and Laplace predictors of the random effects [11,12], several measures of uncertainty have been proposed for performing inference about the random effects in a GLMM. Arguably the two most recognized ones are the unconditional (i.e., neither responses nor random effects are conditioned on) mean squared error of prediction (UMSEP) [13], and the conditional (on the responses from a finite subset of clusters) mean squared error of prediction (CMSEP) [14]. The UMSEP was first explored for the special case of linear mixed models (LMMs) by [13] and [15], where Taylor expansions are used to provide approximations to the UMSEP assuming the variance components are unknown. Similar approaches have since been suggested to approximate UMSEP in GLMMs - see for instance [16,17]. For GLMMs, [14] introduced the CMSEP and derived an estimator of it for a linear combination of fixed and random effects within a given cluster, conditional on the responses in that cluster. While arguments have been provided for favoring either UMSEP or CMSEP, e.g, [6], to date there is no consensus as to which is the more appropriate measure of uncertainty for random effects predictions in GLMMs. Also, when using CMSEP and UMSEP to construct prediction intervals, normality of the *prediction gap* i.e., the difference between the predicted and realized random effect, is commonly assumed in practice, e.g., [18–20].

Most software used to fit GLMMs does not employ UMSEP or CMSEP for quantifying uncertainty of the prediction gap. For example, both the R packages lme4 [21] and glmmTMB [3,12] instead attempt to compute the unconditional variance of the prediction gap, which differs from the UMSEP by a squared bias term, using two different approximations: the lme4 package considers the second derivative (with respect to the random effects) of the joint likelihood of the data and the random effects, while glmmTMB includes an extra correction term to account for estimation of the fixed effects. To construct prediction intervals for the random effects, both packages (again) couple their respective estimators with an assumption of normality of the prediction gap. We refer to such intervals as normal intervals constructed using the glmmTMB/lme4 estimator. We refer the reader to [22,23] among others for instances where such predictions intervals have been used in real GLMM analysis. Furthermore, we acknowledge the recent work of [20] and [24] on asymptotic distributions for random effects in LMMs specifically; these however are not directly applicable to the GLMM i.e., non-normal response, context.

Even though the UMSEP, CMSEP, and unconditional variance of the prediction gap are all distinct methods for quantifying uncertainty in random effects predictions, remarkably their estimators end up being very similar to each other. Also, the coverage probability for prediction intervals constructed using glmmTMB (but not lme4) tend to achieve close to the nominal significance level in practice, when both the number of clusters and cluster sizes in the GLMM are large. This is despite there being no formal consistency results for the estimators of prediction error used by CMSEP, UMSEP, glmmTMB or lme4, as well as no justification for the assumption of normality for the prediction gap. The empirical results are made even more surprising by the recent work of [25], who showed the prediction gap is not in general asymptotically normal, but instead a convolution of a normal distribution and a normal scale-mixture distribution. In this article, we will refer to this contradiction between the strong empirical performance of glmmTMB, and the theoretical results of [25], as the “normality paradox".

### 1.1 A motivating example

To offer a concrete example which illustrates some of the above conceptual/theoretical issues and their implications for GLMM analysis, we consider data for 65 patients from a clinical trial comparing two groups (bolus/lock-out combinations). The data can be found in the cold package in R [26], and contain the number of bolus requests per interval in 12 successive four-hourly intervals following abdominal surgery. Thus, there are 12 observations (time-points) of the number of requests, a discrete count response, for each individual. Preliminary analysis suggests the bolus/lock-out combination the patient belonged to does not contribute to the number of requests. Hence for illustrative purposes, we do not include a group effect, and include time as the only covariate in our analysis. Let $y_{ij}$ denote the number of requests of the *i*th individual at the *j*th period, for *i* = 1 , … , 65 and *j* = 1 , … , 12.

We model this dataset using a GLMM. Assuming the individuals (clusters) are independent, we use a Poisson GLMM with canonical log link function, such that $\ln(\mu_{ij})=\beta_{0}\;+\;\beta_{1}j$ + $b_{i0}\;+\;b_{i1}j$ where $\mu_{ij}$ denotes the conditional mean response and $\beta_{0}$ and $\beta_{1}$ denote a fixed intercept and slope for the time period, *j*, respectively. Independently and identically distributed (i.i.d.) normal random intercepts and random slopes for time are also included, $b_{i}={\left(b_{i0},b_{i1}\right)}^{⊤}\overset{i.i.d.}{∼}N(0,G)$, for some unstructured 2 × 2 random effects covariance matrix.

We are interested in constructing prediction intervals for the random effects of the individuals. Under the GLMM structure above, all individuals have the same design matrix for both their fixed and random effect covariates, namely the 12 × 2 matrix where the first column is a column of ones, and the second column is ${\left(1,\ldots ,12\right)}^{⊤}$. Together with the assumption that the individuals (clusters) are independent, it follows that individuals are exchangeable, since there is no information distinguishing between them. If we work in an unconditional framework then (i.e., neither responses nor random effects are conditioned on), we should expect the prediction gap, i.e., the *difference* between the predictor of the random effects and the true realised random effects, to have the same marginal distribution for individuals. Put another way, lme4 and glmmTMB (both of which aim to estimate the unconditional variance of the prediction gap), and UMSEP should all produce prediction intervals for ${\dot{b}}_{i}$ which have the same length across all individuals. However, CMSEP should produce prediction intervals that differ between individuals (unless the realized responses are identical).

**Fig 1:**
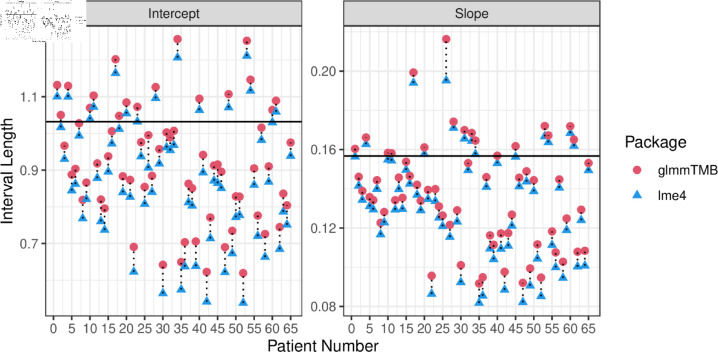
Estimated 95% prediction interval lengths for each individual. Horizontal black line represents the length of the unconditional interval as derived in [25].

Fig 1 presents the results for the random intercepts, $b_{i0}$, and random slopes, $b_{i1}$ of each individual, based on fitting the proposed Poisson GLMM using lme4 and glmmTMB. Note the latter package produces results that are identical to those using the estimators for CMSEP and UMSEP; see Sect 2.1 for a detailed derivation on this. For both packages, the interval lengths for each individual differ substantially; this contradicts the unconditional interpretation that prediction intervals constructed from these packages (as well as from UMSEP) aim to produce. Indeed, the black horizontal line shown in Fig 1 represents results for an unconditional interval derived in [25] based on a normal scale-mixture distribution. These unconditional intervals do not rely on first deriving UMSEP or unconditional variance and then applying a distributional assumption; rather, the bounds are determined directly from the quantiles of the appropriate normal scale-mixture distribution.

It is also worth noting glmmTMB produces consistently wider prediction intervals than lme4, even though both packages use the Laplace approximation to fit the GLMM and obtain almost identical point estimates of the model parameters and predictions of the random effects (see Sect B of the supplementary material). Thus, there a possible implication either glmmTMB is overcovering or lme4 is undercovering. In summary, this simple motivating example raise some interesting questions regarding random effects inference in GLMMs, notably what are these prediction intervals from glmmTMB and lme4 estimating (i.e., what are they consistent for), and how should they be interpreted?

### 1.2 Main contributions

This article aims to resolve the lack of consensus on random effects inference in GLMMs literature, provide a deeper understanding of how uncertainty is quantified and estimated for random effects predictions, and the appropriate way to interpret and use them in practice. The main contributions as are follows:

1. We study proposed estimators of UMSEP and CMSEP for the empirical Bayes and Laplace predictors of the random effects respectively, establishing new connections between these methods for constructing and interpreting prediction inference, and the practical approaches used by glmmTMB and lme4;
2. Leveraging recent theoretical work in [25], we reconcile the normality paradox and offer a formal justification and correct interpretation for the glmmTMB (but not the lme4) approach to constructing prediction intervals of random effects in GLMMs. This is done by deriving novel asymptotic consistency and distributional results for the prediction gap in a sampling framework conditional on a finite subset of the random effects, which is an extension to the results obtained under an unconditional sampling framework in [25];
3. Through a simulation study along with several examples, we offer theoretical justification and discuss practical implications of applying the glmmTMB approach/CMSEP/USMEP coupled with a normality assumption for prediction inference in GLMMs;
4. Focusing particularly on prediction interval lengths, we offer new insights into differences that arise when performing inference in an unconditional framework versus a conditional framework, where the conditioning variables are either the true random effects or the responses of a finite subset of clusters.

The remainder of this article is structured as follows. In Sect 2.1, we define independent cluster GLMMs and discuss two common methods of estimation. Sect 2.2 develops the various measures of prediction uncertainty under consideration. Sect 3.1 introduces consistency and distributional results for the prediction variance estimators used in glmmTMB, and resolve the normality paradox. Sect 3.2 presents results from a simulation study that empirically verify our theoretical developments, and in the Discussion we discuss the implications of our results.

## 2 Materials and methods

### 2.1 Generalized linear mixed models

We study the independent cluster generalized linear mixed model, often applied in longitudinal studies and official statistics for small-area estimation, among other fields. For $j=1,\ldots ,n_{i}$, and *i* = 1 , … , *m*, let $y_{ij}$ denote the *j*th measurement of cluster *i*, $x_{ij}$ denote a vector of $p_{f}$ fixed effect covariates, and $z_{ij}$ denote a vector of $p_{r}$ random effect covariates. Define $N=\sum_{i=1}^{m}n_{i}$ to be the total sample size, $n=m^{-1}N$ to be the mean cluster size, nL= min ⁡ 1≤i≤mni, and nU= max ⁡ 1≤i≤mni, and assume the *m* clusters are independent. Conditional on a $p_{r}$-vector of random effects $b_{i}$, the responses $y_{ij}$ are assumed to be independent observations from the exponential family with mean $\mu_{ij}$ and dispersion parameter *ϕ*. That is, $f(y_{ij}|b_{i};\beta ,\phi )=\exp\;[\phi^{-1}\{y_{ij}{𝜗}_{ij}-a({𝜗}_{ij})\}+c(y_{ij},\phi )]$, for known functions *a* ( ⋅ ) , *c* ( ⋅ ) , such that for a given link function *g* ( ⋅ )  we have $g(\mu_{ij})=g\{a^{\prime }({𝜗}_{ij})\}=\eta_{ij}=x_{ij}^{⊤}\beta +z_{ij}^{⊤}b_{i}$, where $\eta_{ij}$ denotes the linear predictor and *β* denotes a $p_{f}$-vector of unknown fixed effect coefficients. Commonly used distributions within the exponential family include the normal, Poisson, Binomial, and Gamma distributions. The random effects $b_{i}$ are assumed to be independently drawn from a multivariate normal distribution with zero mean vector and unstructured $p_{r}\times p_{r}$ random effects covariance matrix *G*, that is, $b_{i}\overset{i.i.d.}{∼}N(0,G)$.

Let the $n_{i}\;\times \;p_{f}$ matrix $X_{i}={\left[x_{i1},\ldots ,x_{in_{i}}\right]}^{⊤}$, and the $n_{i}\;\times \;p_{r}$ matrix $Z_{i}={\left[z_{i1},\ldots ,z_{in_{i}}\right]}^{⊤}$, so that for each cluster, the mean model for the GLMM can be written as $g(\mu_{i})=X_{i}\beta \;+\;Z_{i}b_{i}$ for $\mu_{i}={\left(\mu_{i1},\ldots ,\mu_{in_{i}}\right)}^{⊤}$, where $g(\mu_{i})={\left\{g(\mu_{i1}),\ldots ,g(\mu_{in_{i}})\right\}}^{⊤}$. We can further concatenate the $g(\mu_{i})$ across clusters and write *g* ( *μ* ) = *Xβ* + *Zb* where $\mu ={\left(\mu_{1}^{⊤},\ldots ,\mu_{m}^{⊤}\right)}^{⊤}$, $X={\left[X_{1}^{⊤},\ldots ,X_{m}^{⊤}\right]}^{⊤}$, $Z=\text{bdiag}(Z_{1},\ldots Z_{m})$, and $b={\left(b_{1}^{⊤},\ldots ,b_{m}^{⊤}\right)}^{⊤}$, where bdiag ( )  denotes the block-diagonal matrix operator. Here, *X* is of dimension $N\times p_{f}$, and *Z* is an $N\times mp_{r}$ sparse block diagonal matrix, with at most $p_{r}$ non-zero components per row.

Let $y_{i}={\left(y_{11},\ldots ,y_{1n_{i}}\right)}^{⊤}$, $y={\left(y_{1}^{⊤},\ldots ,y_{m}^{⊤}\right)}^{⊤}$ and $\psi ={\left\{\beta^{⊤},\phi ,\text{vech}{\left(G\right)}^{⊤}\right\}}^{⊤}$. The marginal log-likelihood function for the independent cluster GLMM is given by


l(ψ):= ln ⁡ f{y;β,ϕ,vech(G)}= ∑i=1m ln ⁡  [∫  {∏j=1nif(yij|bi;β,ϕ)}f{bi;vech(G)}dbi], (1)

where vech ( ⋅ )  denotes the half-vectorization operator which stacks elements from the lower triangular portion of a (symmetric) matrix, column-wise, into a vector. The integral in (1) is not available analytically except in the special case of a normal response with an identity link function i.e., LMMs. Many methods have been devised in order to compute approximate maximum likelihood estimators of the unknown parameters [27–29]. Of these, the most widely used is the Laplace-approximated marginal log-likelihood, which is employed in the R packages lme4 and glmmTMB for fitting GLMMs. This approach is obtained by approximating the integrand of (1) with a quadratic function around its mode. Denote l(b,ψ)=∑ ⁡i=1m ∑j=1ni ln ⁡ f(yij|bi;β,ϕ)+ ∑ ⁡ i=1m ln ⁡ f(bi;G) as the joint log-likelihood function of the responses and random effects, and let *∂* denote a partial derivative and *∇* ⁡  denote a total derivative. The Laplace approximation to the marginal log-likelihood can be derived as


ℓLA(ψ)=l{b(ψ),ψ}−12ln ⁡ det ⁡ [−∂bb⊤ ⁡l{b(ψ),ψ}], (2)

where *b* ( *ψ* )  satisfies $\partial_{b}l\{b(\psi ),\psi \}=0$, i.e., it is the maximizer of *l* ( *b* , *ψ* )  over *b* for a given *ψ*. The Laplace estimates of the model parameters are then obtained as ψ^LA=arg ⁡ max ⁡ ψℓLA(ψ), and Laplace predictors of the random effects as $b({\hat{\psi }}_{LA})$.

A method closely related to the Laplace approximation is penalized quasi-likelihood (PQL) estimation for GLMMs [30]. By treating the random effects covariance matrix *G* as known (or fixed at some value), such that $\psi ={\left(\beta^{⊤},\phi \right)}^{⊤}$, the PQL objective function for an independent cluster GLMM and unknown parameters $𝜃={\left(\beta^{⊤},b^{⊤},\phi \right)}^{⊤}$ is defined as Q(𝜃)=∑ ⁡i=1m ∑j=1ni ln ⁡ f(yij|bi;β,ϕ)−(1∕2)∑ ⁡ i=1mbi⊤ ⁡G−1bi, and the PQL estimator of the fixed effects, random effects and dispersion parameters is defined as 𝜃^=arg ⁡ max ⁡ 𝜃Q(𝜃).

For known *G*, the PQL objective function *Q* ( *𝜃* )  can also be seen as an approximation to the marginal log-likelihood of the GLMM, since ℓPQL(ψ)=∑ ⁡i=1m ∑j=1ni ln ⁡ f{yij|β,bi(ψ),ϕ}−2−1 ∑ ⁡ i=1mbi(ψ)⊤ ⁡G−1bi(ψ)=ℓLA(ψ)+2−1 ln ⁡ det ⁡ [−∂bb⊤ ⁡l{b(ψ),ψ}]+K where *K* is a constant with respect to *ψ*. Then ψ^PQL=arg ⁡ max ⁡ ψℓPQL(ψ). The PQL objective function thus ignores the log-determinant term in the Laplace-approximated marginal log-likelihood (2), which [30] argue is reasonable if the cluster sizes $n_{i}$ are large, since in this case the log-determinant term varies slowly as a function of  ( *β* , *ϕ* ) . Put another way, the PQL objective function is similar to the Laplace-approximated objective function provided all the $n_{i}$’s are sufficiently large and growing. For this reason, although the theoretical results in this article for prediction inference are for the PQL estimator, we can reasonably apply them to the estimator based on the Laplace approximation; see also the work of [31,32] as well as the simulation results of Sect 3.2 which corroborate this point.

The Laplace and PQL procedures both provide estimates of the model parameters (i.e., fixed effects and dispersion parameters), as well as predictors of the random effects. In the latter, it is straightforward to show both produce the modal predictor for the true realized random effects i.e., the predictor is equal to the modal predictor of $f(b|y;\hat{\psi })$, the conditional distribution of the random effects given the observed data evaluated at the estimated model parameter values.

### 2.2 Measures of prediction uncertainty for random effects

In this section, we outline the derivation of three prominent measures of prediction uncertainty in GLMMs: the unconditional and conditional mean squared errors of prediction (UMSEP and CMSEP, respectively), and the unconditional variance of the prediction gap for a given cluster employed by glmmTMB. By synthesizing the existing literature, in Sects 2.2.1-2.2.3 we highlight common steps in the derivation of all three approaches as well as steps where assumptions are made without formal justification. In doing so, we show that although the aims are different for these three measures, the final estimators of each measure are similar and sometimes even identical in form. In Sect 2.2.4, we also discuss the measure of prediction uncertainty used by lme4, and how it relates to the aforementioned three measures.

Let $\dot{\psi }$ denote the true parameter values of the GLMM, $b(\psi )={\left\{b_{1}(\psi ),\ldots ,b_{m}(\psi )\right\}}^{⊤}$, $\hat{\psi }$ denote the estimates of the model parameters obtained from either Laplace or PQL estimation, and ${\dot{b}}_{i}$ denote the true realized random effect for the *i*th cluster. The CMSEP, UMSEP and glmmTMB uncertainty are defined as $E_{(\dot{b},y)|y_{i}}[\{b_{i}(\hat{\psi })\;-\;{\dot{b}}_{i}\}{\left\{b_{i}(\hat{\psi })\;-\;{\dot{b}}_{i}\right\}}^{⊤}]$, $E_{\dot{b},y}[\{b(\hat{\psi })\;-\;\dot{b}\}{\left\{b(\hat{\psi })\;-\;\dot{b}\right\}}^{⊤}]$, and $V_{\dot{b},y}\{b(\hat{\psi })\;-\;\dot{b}\}$ respectively. The derivations of the estimators of all three measures follow a similar general approach: all are decomposed into two terms with one representing the uncertainty in prediction if the model parameters are known, and another representing the uncertainty from having to estimate the model parameters. A Taylor expansion of $b(\hat{\psi })$ around $\dot{\psi }$ is applied then to the second term. Finally, estimators are proposed for the resulting approximations.

Two further remarks are worth making regarding the derivation of all three measures. First, a common assumption made is that the distribution of the random effects conditional on the observed data is multivariate normal. This implies the mean and median of the random effects given the observed data are equal. Asymptotic equality of the mean and median of the true random effects given the observed data can also be rationalized without this multivariate normal assumption, if cluster sizes are growing. A second assumption common to all three derivations is that the conditional variance of the random effects given the observed data is given by the inverse of the second derivative of the joint likelihood of the responses and random effects. This assumption is often rationalized by appealing to the Laplace approximation to the (marginal) likelihood, which also relies on growing cluster sizes. To our knowledge, there are no formal results in the literature justifying either of these assumptions.

We introduce some notation used in the derivations below. Let $b_{i}(\psi )$, which is a function of both parameters *ψ* and observed data $y_{i}$, denote a predictor of ${\dot{b}}_{i}$. By construction, the ${\hat{b}}_{i}$ from either the PQL or Laplace procedure in Sect 2.1 can be obtained as arg ⁡ max ⁡ bili(b,ψ)=arg ⁡ max ⁡ bi∑ ⁡j=1ni ln ⁡ f(yij|bi;β,ϕ)+ln ⁡ f(bi;G) for any given *ψ*. Thus, they may also be written as $b_{i}(\hat{\psi })$ where $b_{i}(\psi )$ is the mode of $f({\dot{b}}_{i}|y_{i};\psi )$ and $\hat{\psi }$ can be either ${\hat{\psi }}_{PQL}$ or ${\hat{\psi }}_{LA}$. Lastly, recall l(b,ψ)=∑ ⁡i=1m ∑j=1ni ln ⁡ f(yij|bi;β,ϕ)+∑ ⁡ i=1m ln ⁡ f(bi;G) denotes the joint log-likelihood of the data and random effects, and *l* ( *ψ* )  denotes the marginal log-likelihood given by (1).

#### 2.2.1 CMSEP

We begin by deriving an estimator of CMSEP, based on the work of [14]. For GLMMs, [14] advocated inference should be performed conditional on the observed responses belonging to the same clusters as the random effects of interest. That is, they suggested and studied the quantity $E_{(\dot{b},y)|y_{i}}\{{\left({\hat{\eta }}_{i}^{*}\;-\;{\dot{\eta }}_{i}^{*}\right)}^{2}\}$, which is the mean squared prediction error of the linear predictor $\eta_{i}^{*}=x^{*⊤}\beta \;+\;z^{*⊤}b_{i}$ for a particular cluster and some $x^{*}$ and $z^{*}$, conditional on the observations in that cluster. To better highlight the connection between CMSEP and the UMSEP/glmmTMB derivations later, we will apply the same steps as their derivation to a multivariate version of the CMSEP, specifically, the quantity $E_{(\dot{b},y)|y_{i}}[\{b_{i}(\hat{\psi })-{\dot{b}}_{i}\}{\left\{b_{i}(\hat{\psi })-{\dot{b}}_{i}\right\}}^{⊤}]$.

We begin by writing


$$\begin{array}{llll} & E_{(\dot{b},y)|y_{i}}[\{b_{i}(\hat{\psi })-{\dot{b}}_{i}\}{\left\{b_{i}(\hat{\psi })-{\dot{b}}_{i}\right\}}^{⊤}]\; & & \;\\ & =E_{(\dot{b},y)|y_{i}}[\{b_{i}(\hat{\psi })-b_{i}(\dot{\psi })+b_{i}(\dot{\psi })-{\dot{b}}_{i}\}{\left\{b_{i}(\hat{\psi })-b_{i}(\dot{\psi })+b_{i}(\dot{\psi })-{\dot{b}}_{i}\right\}}^{⊤}].\; & & \; \end{array}$$

Assume $E_{{\dot{b}}_{i}|y_{i}}({\dot{b}}_{i})=b_{i}(\dot{\psi })$, i.e., the predictor for ${\dot{b}}_{i}$ is the mean of the random effect given the data. Note for the modal predictor considered by [12] for glmmTMB and [16] for UMSEP, this assumption holds if $f({\dot{b}}_{i}|y_{i})$ is multivariate normal. On the other hand, [14] explicitly advocated using $b_{i}(\dot{\psi })=E_{{\dot{b}}_{i}|y_{i}}({\dot{b}}_{i})$ instead of the modal predictor in GLMMs. Since $b_{i}(\hat{\psi })\;-\;b_{i}(\dot{\psi })$ and $b_{i}(\dot{\psi })\;-\;{\dot{b}}_{i}$ are conditionally independent given $y_{i}$, the CMSEP can be further rewritten as


$$\begin{array}{llll} E_{(\dot{b},y)|y_{i}}[\{b_{i}(\hat{\psi })-{\dot{b}}_{i}\}{\left\{b_{i}(\hat{\psi })-{\dot{b}}_{i}\right\}}^{⊤}] & =E_{(\dot{b},y)|y_{i}}[\{b_{i}(\dot{\psi })-{\dot{b}}_{i}\}{\left\{b_{i}(\dot{\psi })-{\dot{b}}_{i}\right\}}^{⊤}]\; & & \;\\ & +E_{(\dot{b},y)|y_{i}}[\{b_{i}(\hat{\psi })-b_{i}(\dot{\psi })\}{\left\{b_{i}(\hat{\psi })-b_{i}(\dot{\psi })\right\}}^{⊤}]\; & & \;\\ & \overset{\Delta }{=}C_{1}+C_{2}.\; & & \; \end{array}$$

The two terms in the decomposition may be thought of as encapsulating the naive prediction variance arising from predicting for $\dot{b}$ when $\dot{\psi }$ is known, plus an adjustment term for needing to estimate $\dot{\psi }$. Again using $E_{{\dot{b}}_{i}|y_{i}}({\dot{b}}_{i})=b_{i}(\dot{\psi })$, we have $C_{1}=V_{{\dot{b}}_{i}|y_{i}}({\dot{b}}_{i})$. Also, from the Laplace approximation [27] we know $-{\left[\partial_{b_{i}{b_{i}}^{⊤}}l_{i}\{b_{i}(\dot{\psi }),\dot{\psi }\}\right]}^{-1}$ approximates this conditional variance with a relative error of $O_{p}(n_{i}^{-1})$. Thus, we can estimate $C_{1}$ by $-{\left[\partial_{b_{i}{b_{i}}^{⊤}}l_{i}\{b_{i}(\hat{\psi }),\hat{\psi }\}\right]}^{-1}$, provided $\hat{\psi }$ is a consistent estimator (conditional on $y_{i}$) of $\dot{\psi }$.

Turning to $C_{2}$, we have from a Taylor expansion of $b(\hat{\psi })$ around $\dot{\psi }$ that


$$\begin{array}{ccc} b(\hat{\psi })-b(\dot{\psi })=\partial_{\psi^{⊤}}b(\dot{\psi })(\hat{\psi }-\dot{\psi })+O_{p}(m^{-1}), & & \end{array}$$ (3)

where the order of the error term on the right hand side is shown in [33]. Since by definition $\partial_{b}l\{b(\psi ),\psi \}=0$ for any *ψ*, by the multivariate chain rule we obtain $\nabla_{\psi^{⊤}}\partial_{b}l\{b(\psi ),\psi \}=\partial_{bb^{⊤}}l\{b(\psi ),\psi \}\partial_{\psi^{⊤}}b(\psi )+\partial_{\psi^{⊤}}\partial_{b}l\{b(\psi ),\psi \}=0$, and thus


$$\begin{array}{ccc} \partial_{\psi^{⊤}}b(\dot{\psi })=-{\left[\partial_{bb^{⊤}}l\{b(\dot{\psi }),\dot{\psi }\}\right]}^{-1}\partial_{b\psi^{⊤}}l\{b(\dot{\psi }),\dot{\psi }\}. & & \end{array}$$ (4)

By substituting Eq (4) into Eq (3) and noting $\partial_{bb^{⊤}}l\{b(\hat{\psi }),\hat{\psi }\}$ is block-diagonal, we obtain


$$\begin{array}{llll} C_{2} & =E_{(\dot{b},y)|y_{i}}[\{b_{i}(\hat{\psi })-b_{i}(\dot{\psi })\}{\left\{b_{i}(\hat{\psi })-b_{i}(\dot{\psi })\right\}}^{⊤}]\; & & \;\\ & ={\left[\partial_{b_{i}{b_{i}}^{⊤}}l_{i}\{b_{i}(\dot{\psi }),\dot{\psi }\}\right]}^{-1}\partial_{b_{i}\psi^{⊤}}l_{i}\{b_{i}(\dot{\psi }),\dot{\psi }\}E_{(\dot{b},y)|y_{i}}[(\hat{\psi }-\dot{\psi })\; & & \;\\ & {\left(\hat{\psi }-\dot{\psi }\right)}^{⊤}]{\left[\partial_{b_{i}\psi^{⊤}}l_{i}\{b_{i}(\dot{\psi }),\dot{\psi }\}\right]}^{⊤}{\left[\partial_{b_{i}{b_{i}}^{⊤}}l_{i}\{b_{i}(\dot{\psi }),\dot{\psi }\}\right]}^{-1}+O(m^{-1}),\; & & \; \end{array}$$

where the second equation follows because ${\left[\partial_{b_{i}{b_{i}}^{⊤}}l_{i}\{b_{i}(\dot{\psi }),\dot{\psi }\}\right]}^{-1}\partial_{b_{i}\psi^{⊤}}l_{i}\{b_{i}(\dot{\psi }),\dot{\psi }\}$ is not a function of ${\dot{b}}_{i}$ or $y_{-i}$, the $\left(N\;-\;n_{i}\right)$-vector formed by deleting $y_{i}$ from *y*. Since the conditional (on *y_i_*) and unconditional variance of $\hat{\psi }\;-\;\dot{\psi }$ are in agreement to order $O_{p}(m^{-1})$ [14], if we assume $E_{(\dot{b},y)|y_{i}}(\hat{\psi }-\dot{\psi })=0$ we may subsequently estimate $C_{2}$ by estimating ${\left[\partial_{b_{i}{b_{i}}^{⊤}}l_{i}\{b_{i}(\dot{\psi }),\dot{\psi }\}\right]}^{-1}\partial_{b_{i}\psi^{⊤}}l_{i}\{b_{i}(\dot{\psi }),\dot{\psi }\}V_{y}(\hat{\psi }){\left[\partial_{b_{i}\psi^{⊤}}l_{i}\{b_{i}(\dot{\psi }),\dot{\psi }\}\right]}^{⊤}{\left[\partial_{b_{i}{b_{i}}^{⊤}}l_{i}\{b_{i}(\dot{\psi }),\dot{\psi }\}\right]}^{-1}$. Furthermore, from standard results on maximum likelihood estimation, we can approximate $V_{y}(\dot{\psi })$ by ${\left\{-\nabla_{\psi \psi^{⊤}}\ell_{PQL}(\dot{\psi })\right\}}^{-1}$ or ${\left\{-\nabla_{\psi \psi^{⊤}}\ell_{LA}(\dot{\psi })\right\}}^{-1}$, after which we obtain the estimate ${\hat{V}}_{y}(\hat{\psi })$ by replacing $\dot{\psi }$ with $\hat{\psi }$. This estimate is reasonable provided both the number of clusters (in order to appeal to standard maximum likelihood asymptotics e.g., [34]) and the cluster sizes (to guarantee the PQL/Laplace approximation is sufficiently accurate for true marginal log-likelihood) grow.

Combining the estimators for $C_{1}$ and $C_{2}$, we obtain the estimator for CMSEP,


$$\begin{array}{llll} & {\hat{V}}_{CMSEP}\{b_{i}(\hat{\psi })-{\dot{b}}_{i}\}=-{\left[\partial_{b_{i}{b_{i}}^{⊤}}l_{i}\{b_{i}(\hat{\psi }),\hat{\psi }\}\right]}^{-1}+\; & & \;\\ & {\left[\partial_{b_{i}{b_{i}}^{⊤}}l_{i}\{b_{i}(\hat{\psi }),\hat{\psi }\}\right]}^{-1}\partial_{b_{i}\psi^{⊤}}l_{i}\{b_{i}(\hat{\psi }),\hat{\psi }\}V_{y}(\hat{\psi }){\left[\partial_{b_{i}\psi^{⊤}}l_{i}\{b_{i}(\hat{\psi }),\hat{\psi }\}\right]}^{⊤}{\left[\partial_{b_{i}{b_{i}}^{⊤}}l_{i}\{b_{i}(\hat{\psi }),\hat{\psi }\}\right]}^{-1}\; & \text{(5)}\; \end{array}$$

**Remark 1**. *[14] propose a bootstrap procedure to adjust for the error introduced when replacing* $\dot{\psi }$ *with* $\hat{\psi }$*, which is non-negligible to second order when the cluster sizes are fixed. However, this error is negligible when both the number of clusters and cluster sizes are allowed to grow. We will see later that without the bootstrap adjustment, the CMSEP and* glmmTMB *and UMSEP estimators are identical.*

We point out two key aspects regarding Eq (5):

1. [14] do not actually show (5) is a consistent estimator of the true CMSEP. That is, there is no proof ${\hat{V}}_{CMSEP}\{b_{i}(\hat{\psi })-{\dot{b}}_{i}\}-E_{(\dot{b},y)|y_{i}}[\{b_{i}(\hat{\psi })-{\dot{b}}_{i}\}{\left\{b_{i}(\hat{\psi })-{\dot{b}}_{i}\right\}}^{⊤}]\}=o_{p}(E_{(\dot{b},y)|y_{i}}[\{b_{i}(\hat{\psi })-{\dot{b}}_{i}\}{\left\{b_{i}(\hat{\psi })-{\dot{b}}_{i}\right\}}^{⊤}])$.
2. To construct prediction intervals for ${\dot{b}}_{i}$ using the estimated CMSEP, we require a distributional result. For example, it turns out the package glmmTMB uses () as if it were a variance, and assumes normality of $b(\hat{\psi })\;-\;\dot{b}$. Using (5) as if it were a variance and assuming normality would require exact or asymptotic normality of $b(\hat{\psi })\;-\;\dot{b}$ conditional on $y_{i}$; to our knowledge this has not been proven in the literature (see Section 3.1 for further discussion).

#### 2.2.2 UMSEP

While there are several results on the UMSEP for linear predictors in LMMs [13,15,35], few such results exists for GLMMs. Two notable exceptions are the works of [16,17], who consider logistic link binomial GLMMs and estimate the UMSEP by considering a Taylor linearization of the mean responses in the linear predictor, before applying the LMM results of [15]. By contrast, the derivation of UMSEP we develop below more closely follows the work of [13], which does not adjust for the error introduced when replacing $\dot{\psi }$ with $\hat{\psi }$; this error is negligible if both the number of clusters and cluster sizes are allowed to grow.

For UMSEP, the goal is to estimate the quantity $E_{\dot{b},y}[\{b(\hat{\psi })-\dot{b}\}{\left\{b(\hat{\psi })-\dot{b}\right\}}^{⊤}]$. From the law of iterated expectation, we have


$$\begin{array}{llll} E_{\dot{b},y}[\{b(\hat{\psi })-\dot{b}\}{\left\{b(\hat{\psi })-\dot{b}\right\}}^{⊤}] & =E_{y}E_{\dot{b}|y}[\{b(\dot{\psi })-\dot{b}\}{\left\{b(\dot{\psi })-\dot{b}\right\}}^{⊤}]\; & & \;\\ & \;+E_{y}E_{\dot{b}|y}[\{b(\hat{\psi })-b(\dot{\psi })\}{\left\{b(\hat{\psi })-b(\dot{\psi })\right\}}^{⊤}]\; & & \;\\ & \overset{\Delta }{=}U_{1}+U_{2},\; & & \; \end{array}$$

where in the second line we have assumed $b(\dot{\psi })=E_{\dot{b}|y}(\dot{b})$. As in the derivation for CMSEP, this equality holds if $f(\dot{b}|y)$ is (assumed to be) multivariate normally distributed. As an aside, [13] require the estimator of the random effects covariance parameters to be translation-invariant in order for the cross-product terms to be zero. We avoid this requirement as we consider the UMSEP of the prediction gap as opposed to the linear predictor.

Next, making the assumption $b(\dot{\psi })=E_{\dot{b}|y}(\dot{b})$ again we have $U_{1}=E_{y}V_{\dot{b}|y}(\dot{b})$, which we subsequently estimate using ${\left\{-\partial_{bb^{⊤}}l(b(\hat{\psi }),\hat{\psi })\right\}}^{-1}$. Note an equivalent term also appears in the derivation of UMSEP in [16,17], and is estimated in essentially the same way.

Turning to $U_{2}$, we first note the inner expectation disappears because $b(\hat{\psi })\;-\;b(\dot{\psi })$ conditional on *y* is not a function of $\dot{b}$. Next, we make use of the Taylor expansion of $b(\hat{\psi })$ around $\dot{\psi }$ i.e., using Eqs (3) and (4), we obtain


$$\begin{array}{llll} U_{2} & =E_{y}({\left[\partial_{bb^{⊤}}l\{b(\dot{\psi }),\dot{\psi }\}\right]}^{-1}\partial_{b\psi^{⊤}}l\{b(\dot{\psi }),\dot{\psi }\}(\hat{\psi }-\dot{\psi })\; & & \;\\ & {\left(\hat{\psi }-\dot{\psi }\right)}^{⊤}{\left[\partial_{b\psi^{⊤}}l\{b(\dot{\psi }),\dot{\psi }\}\right]}^{⊤}{\left[\partial_{bb^{⊤}}l\{b(\dot{\psi }),\dot{\psi }\}\right]}^{-1}+O_{p}(m^{-1})).\; & & \; \end{array}$$

Next, we draw a parallel with the work of [13] and make the following approximations, which are discussed in Sect E of the supplementary material. Assume the smaller order term has finite expectation, treat *b* ( *ψ* )  as if it was not a function of *y*, and assume $E_{\dot{b},y}(\hat{\psi }\;-\;\dot{\psi })=0$. By approximating $E_{y}\{(\hat{\psi }-\dot{\psi }){\left(\hat{\psi }-\dot{\psi }\right)}^{⊤}\}$ with $V_{y}(\hat{\psi })$, we may then approximate $U_{2}$ directly by


$$\begin{array}{lll} U_{3}={\left[\partial_{bb^{⊤}}l\{b(\dot{\psi }),\dot{\psi }\}\right]}^{-1}\partial_{b\psi^{⊤}}l\{b(\dot{\psi }),\dot{\psi }\}V_{y}(\hat{\psi }){\left[\partial_{b\psi^{⊤}}l\{b(\dot{\psi }),\dot{\psi }\}\right]}^{⊤}{\left[\partial_{bb^{⊤}}l\{b(\dot{\psi }),\dot{\psi }\}\right]}^{-1}, & \; & \end{array}$$

where, similar to the CMSEP derivation, $V_{y}(\hat{\psi })$ is approximated by ${\left\{-\nabla_{\psi \psi^{⊤}}\ell_{PQL}(\dot{\psi })\right\}}^{-1}$ or ${\left\{-\nabla_{\psi \psi^{⊤}}\ell_{LA}(\dot{\psi })\right\}}^{-1}$. Finally, by replacing $\dot{\psi }$ with $\hat{\psi }$, and combining terms $U_{1}$ and $U_{3}$ and focusing on the the *i*th cluster, we obtain the UMSEP estimator


$$\begin{array}{llll} & {\hat{V}}_{UMSEP}\{b_{i}(\hat{\psi })-{\dot{b}}_{i}\}=-{\left[\partial_{b_{i}{b_{i}}^{⊤}}l_{i}\{b_{i}(\hat{\psi }),\hat{\psi }\}\right]}^{-1}+\; & & \;\\ & {\left[\partial_{b_{i}{b_{i}}^{⊤}}l_{i}\{b_{i}(\hat{\psi }),\hat{\psi }\}\right]}^{-1}\partial_{b_{i}\psi^{⊤}}l_{i}\{b_{i}(\hat{\psi }),\hat{\psi }\}V_{y}(\hat{\psi }){\left[\partial_{b_{i}\psi^{⊤}}l_{i}\{b_{i}(\hat{\psi }),\hat{\psi }\}\right]}^{⊤}{\left[\partial_{b_{i}{b_{i}}^{⊤}}l_{i}\{b_{i}(\hat{\psi }),\hat{\psi }\}\right]}^{-1},\; & & \; \end{array}$$

where we note $\partial_{bb^{⊤}}l\{b(\hat{\psi }),\hat{\psi }\}$ is block-diagonal in structure. It is not hard to see the resulting estimator of UMSEP is identical to the CMSEP estimator.

**Remark 2**. *Since*


$$\begin{array}{lll} E_{\dot{b},y}[\{b_{i}(\hat{\psi })-{\dot{b}}_{i}\}{\left\{b_{i}(\hat{\psi })-{\dot{b}}_{i}\right\}}^{⊤}]=E_{y_{i}}E_{(\dot{b},y)|y_{i}}[\{b_{i}(\hat{\psi })-{\dot{b}}_{i}\}{\left\{b_{i}(\hat{\psi })-{\dot{b}}_{i}\right\}}^{⊤}], & \; & \end{array}$$

*then one may view the CMSEP as a ‘central approximation’ [12] for the UMSEP, and in this sense it is not surprising the estimators are the same. Indeed, approximating* $U_{1}$ *by its central approximation (i.e., approximating an expectation by a single realization of a random variable with that expectation), and treating* *b* ( *ψ* )  *as if it is not a function of* *y* *in the approximation to* $U_{2}$*, are the steps that make the UMSEP estimator the same as the CMSEP estimator. Thus one way to view the estimator is as a central approximation of the true UMSEP.*

In seeking to apply the estimator for the UMSEP as the basis for constructing prediction intervals for random effects in GLMMs, we again run into the same points faced by the estimator for CMSEP. That is, to our knowledge there is no consistency result for the UMSEP estimator, and neither is there a distributional result for the prediction gap.

#### 2.2.3 The glmmTMB estimator

We follow the work of [12] and [33] to derive the estimator used in the glmmTMB package for the quantity $V_{\dot{b},y}(b(\hat{\psi })-\dot{b})$ i.e., the unconditional variance of the prediction gap $b(\hat{\psi })-\dot{b}$, where $\hat{\psi }$ can be either ${\hat{\psi }}_{LA}$ or ${\hat{\psi }}_{PQL}$. From henceforth, we will also refer to this estimator simply as the glmmTMB estimator.

To begin, from the law of total variance we have


$$\begin{array}{lll} V_{\dot{b},y}\{b(\hat{\psi })-\dot{b}\}=E_{y}V_{\dot{b}|y}\{b(\hat{\psi })-\dot{b}\}+V_{y}E_{\dot{b}|y}\{b(\hat{\psi })-\dot{b}\}\overset{\Delta }{=}T_{1}+T_{2}. & \; & \end{array}$$

We replace $T_{1}=E_{y}V_{\dot{b}|y}\{b(\hat{\psi })-\dot{b}\}$ by its central approximation $V_{\dot{b}|y}\{b(\hat{\psi })-\dot{b}\}$ - see [12] as well as Remark 2 above. Similarly to UMSEP, this step has no formal justification, and we will see in Sect 3.1 that this step prevents the final estimator from being a consistent estimator of $V_{\dot{b},y}\{b(\hat{\psi })\;-\;\dot{b}\}$ in general. Next, identical to $U_{1}$ in the derivation of the UMSEP, we approximate $V_{\dot{b}|y}\{b(\hat{\psi })\;-\;\dot{b}\}=V_{\dot{b}|y}(\dot{b})$ by ${\left[-\partial_{bb^{⊤}}l\{b(\dot{\psi }),\dot{\psi }\}\right]}^{-1}$. From this, a reasonable estimator of $T_{1}$ is thus given by ${\left[-\partial_{bb^{⊤}}l\{b(\hat{\psi }),\hat{\psi }\}\right]}^{-1}$, provided $\hat{\psi }$ is a consistent estimator for $\dot{\psi }$. This is then the same estimator as for *U*_1_ in the UMSEP, which is expected since $U_{1}=T_{1}$ when $E(\hat{\psi }-\dot{\psi })=0$. It is also analogous to $C_{1}$ in the derivation of the CMSEP, in the sense the *i*th-diagonal block of ${\left[-\partial_{bb^{⊤}}l\{b(\hat{\psi }),\hat{\psi }\}\right]}^{-1}$ is the estimator for $C_{1}$.

Turning to $T_{2}$, write $T_{2}=V_{y}\{b(\hat{\psi })-E_{\dot{b}|y}(\dot{b})\}$. Assuming $b(\dot{\psi })=E_{\dot{b}|y}(\dot{b})$ is used, then $T_{2}=V_{y}\{b(\hat{\psi })-b(\dot{\psi })\}$. By applying Eqs (3)–(4) once more, we obtain $T_{2}=V_{y}(-{\left[\partial_{bb^{⊤}}l\{b(\dot{\psi }),\dot{\psi }\}\right]}^{-1}\partial_{b\psi^{⊤}}l\{b(\dot{\psi }),\dot{\psi }\}(\hat{\psi }-\dot{\psi })+O_{p}(m^{-1}))$, which, by treating $b(\dot{\psi })$ as if it is not a function of *y*, can be further approximated as


$$\begin{array}{lll} {\left[\partial_{bb^{⊤}}l\{b(\dot{\psi }),\dot{\psi }\}\right]}^{-1}\partial_{b\psi^{⊤}}l\{b(\dot{\psi }),\dot{\psi }\}V_{y}(\hat{\psi }){\left[\partial_{b\psi^{⊤}}l\{b(\dot{\psi }),\dot{\psi }\}\right]}^{⊤}{\left[\partial_{bb^{⊤}}l\{b(\dot{\psi }),\dot{\psi }\}\right]}^{-1}≜T_{22}=U_{3}. & \; & \end{array}$$

[33] show that when $b(\hat{\psi })$ is the Laplace predictor, $T_{2}=T_{22}\;+\;O_{p}(m^{-3∕2})$, which justifies this approximation. Note the estimator for $T_{2}$ is, again, similar to the estimator for $C_{2}$ in the derivation of the CMSEP: the latter is a sub-matrix of the former due to the block-diagonality of $\partial_{bb^{⊤}}l{\left\{b(\hat{\psi }),\hat{\psi }\right\}}^{-1}$.

On combining the estimators for $T_{1}$ and $T_{2}$ above, we obtain ${\hat{V}}_{\dot{b},y}\{b(\hat{\psi })\;-\;\dot{b}\}=-\partial_{bb^{⊤}}l{\left\{b(\hat{\psi }),\hat{\psi }\right\}}^{-1}\;+\;\partial_{bb^{⊤}}l\{b(\hat{\psi }),\hat{\psi }\}\partial_{b\psi^{⊤}}l\{b(\hat{\psi }),\hat{\psi }\}{\hat{V}}_{y}(\hat{\psi })\partial_{b^{⊤}\psi }l\{b(\hat{\psi })$, $\hat{\psi }\}\partial_{bb^{⊤}}l\{b(\hat{\psi }),\hat{\psi }\}$. Finally, to obtain the estimated covariance matrix of ${\hat{b}}_{i}-{\dot{b}}_{i}$, we can extract the corresponding sub-matrix, which since $\partial_{bb^{⊤}}l\{b(\hat{\psi }),\hat{\psi }\}$ is block-diagonal, this gives


$$\begin{array}{llll} & {\hat{V}}_{\dot{b},y}\{b_{i}(\hat{\psi })-{\dot{b}}_{i}\}=-{\left[\partial_{b_{i}{b_{i}}^{⊤}}l_{i}\{b_{i}(\hat{\psi }),\hat{\psi }\}\right]}^{-1}+\; & & \;\\ & {\left[\partial_{b_{i}{b_{i}}^{⊤}}l_{i}\{b_{i}(\hat{\psi }),\hat{\psi }\}\right]}^{-1}\partial_{b_{i}\psi^{⊤}}l_{i}\{b_{i}(\hat{\psi }),\hat{\psi }\}V_{y}(\hat{\psi }){\left[\partial_{b_{i}\psi^{⊤}}l_{i}\{b_{i}(\hat{\psi }),\hat{\psi }\}\right]}^{⊤}{\left[\partial_{b_{i}{b_{i}}^{⊤}}l_{i}\{b_{i}(\hat{\psi }),\hat{\psi }\}\right]}^{-1}.\; & \text{(6)}\; \end{array}$$

Note this is identical to the both the estimators of the CMSEP, ${\hat{V}}_{CMSEP}\{b_{i}(\hat{\psi })-{\dot{b}}_{i}\}$ in Eq (5), and the UMSEP, ${\hat{V}}_{UMSEP}\{b_{i}(\hat{\psi })-{\dot{b}}_{i}\}$.

**Remark 3**. *The* $E_{y}(\hat{\psi }-\dot{\psi })=0$ *assumption is the key step that makes the* glmmTMB *estimator (6) identical to the UMSEP estimator. It is not surprising these two estimators are identical because*


$$\begin{array}{llll} E_{\dot{b},y}[\{b(\hat{\psi })-\dot{b}\}{\left\{b(\hat{\psi })-\dot{b}\right\}}^{⊤}] & =V_{\dot{b},y}\{b(\hat{\psi })-\dot{b}\}+E_{\dot{b},y}\{b(\hat{\psi })-\dot{b}\}E_{\dot{b},y}{\left\{b(\hat{\psi })-\dot{b}\right\}}^{⊤},\; & & \; \end{array}$$

*and [33] show* $E_{\dot{b},y}\{b(\hat{\psi })-\dot{b}\}E_{\dot{b},y}{\left\{b(\hat{\psi })-\dot{b}\right\}}^{⊤}=O(m^{-1})$. *Thus UMSEP and the unconditional variance of the prediction gap only differ by a smaller order term, so they can be estimated in the same way when the number of clusters is large.*

The glmmTMB estimator suffers the same two critical problems as the estimators for the CMSEP and the UMSEP estimators: to our knowledge there is no consistency result for ${\hat{V}}_{\dot{b},y}\{b_{i}(\hat{\psi })-{\dot{b}}_{i}\}$, and neither is there a distributional result for the prediction gap.

#### 2.2.4 lme4

Finally, we consider the approach taken by the lme4 package, which estimates the unconditional variance of the prediction gap using ${\left[-\partial_{bb^{⊤}}l\{b(\hat{\psi }),\hat{\psi }\}\right]}^{-1}$. That is, it uses only the first part of the glmmTMB estimator in (5). [12] claimed the estimator for term $T_{2}$, the second part of the glmmTMB estimator, is negligible in large samples due to the assumed consistency of $\hat{\psi }$. However, this statement is only true if the number of clusters *m* grows while all cluster sizes $n_{i}$ are fixed. On the other hand, growing cluster sizes are required for the consistency of the PQL or Laplace estimators, the latter of which is used for estimation by glmmTMB.

In Sects 3.1 and 3.2, we show the lme4 estimator is, in general, not an adequate measure of prediction uncertainty when the cluster sizes are larger than the number of clusters, and can result in substantial undercoverage when used as the basis for constructing prediction intervals. Indeed, the omission of the second term of the glmmTMB estimator causes the noticeable differences in the prediction interval lengths between the packages lme4 and glmmTMB (as exemplified in Fig 1 shown earlier).

## 3 Results

### 3.1 Normality paradox

To construct prediction intervals for the random effects in GLMMs, both the glmmTMB and lme4 packages assume the prediction gap $b(\hat{\psi })\;-\;\dot{b}$ is multivariate normally distributed with mean vector zero and covariance matrix given by their respective estimators e.g., Eq (5) in the case of glmmTMB. The assumption of normality is also used when using CMSEP and UMSEP to build prediction intervals, e.g., [18,19]. However, as discussed throughout Sect 2.2 there is no formal justification for this assumption under GLMMs. Indeed, recent research by [25] shows the prediction gap is, in general, not asymptotically normal: under an unconditional framework when both *m* and $n_{i}$ grow, and if the random and fixed effects have the same covariates i.e., are *partnered*, they prove that the prediction gap derived from the PQL estimator converges in distribution to a convolution of a normal distribution and a normal scale-mixture distribution; see Sect 3.1.2 for a formal definition of this. Recall also this result was applied to obtain the horizontal solid black line in Fig 1, from which it is apparent the unconditional distribution of the prediction gap can yield markedly different prediction intervals compared to glmmTMB and lme4.

The results from [25] call into question the justification for assuming normality as the basis for constructing prediction intervals. Despite this however, in practice (e.g., see the simulation study in Sect 3.2) the prediction intervals constructed using the glmmTMB estimator and assuming normality actually achieve close to nominal coverage when both the number of clusters and cluster sizes in the GLMM are large. As such, there seems to be a contradiction between empirical performance (which shows assuming normality for the prediction gap results in reasonable performance), and the theoretical results in [25] (which show this is asymptotically incorrect).

In this next subsection, we reconcile the above “normality paradox" by providing a formal justification for using the glmmTMB estimator and assuming normality of the prediction gap to construct intervals for the random effects. In doing so, we also resolve an issue identified in our motivating example in Sect 1.1, namely that the prediction interval lengths from glmmTMB differ across clusters even when the clusters are exchangeable. This contradicts the stated goal of the glmmTMB estimator as discussed in see Sect 2.2.3, which is to estimate the unconditional variance of the prediction gap and produce unconditional intervals. We develop our asymptotic results for the PQL estimator, although in Sect 3.2 we demonstrate empirically that they also work for the Laplace estimator when *m* and $n_{i}$ grow.

#### 3.1.1 Technical results

We consider independent cluster GLMMs, and make the following additional simplifying assumptions.

(S1) $X_{i}=Z_{i}$ for *i* = 1 , … , *m*. That is, all covariates included as fixed effects covariates are also included as random effects covariates and vice versa. Note in this partnered case, $p_{f}=p_{r}=:p$.
(S2) The canonical link function is used, so that ${𝜗}_{ij}=\eta_{ij}$ for $j=1,\ldots ,n_{i}$ and *i* = 1 , … , *m*.
(S3) $n_{i}=O(n_{L})$ uniformly for *i* = 1 , … , *m*, and the number of clusters and minimum cluster size satisfy $m,n_{L}\to \infty$, and $mn_{L}^{-2}\to 0$.
(S4) The random effects covariance matrix $\dot{G}$ and dispersion parameter $\dot{\phi }$ are known, such that *ψ* = *β* and $𝜃={\left(\beta^{⊤},b^{⊤}\right)}^{⊤}$.

Assumptions (S1)-(S4) are also made in [25]. Assumption (S1), while restrictive, is relevant in practice where, due to a lack of knowledge on which random effects should be included in the model, a fully partnered model is often first fitted [36]. Assumption (S2) is often assumed in GLMM asymptotics, e.g., [32,37]. Assumption (S3) requires cluster sizes to grow at the same rate when deriving asymptotics of the PQL estimator (see also [31]), while the number of clusters cannot grow too fast relative to the cluster size. Finally, assumption (S4) does not detract from the arguments we make in this article given our main focus is on the interpretation of prediction intervals for the random effects. Moreover, it is possible to relax this assumption to employ a working *G* and *ϕ* analogous to [25], but we leave this extension as an avenue of future research.

We note that although very small cluster sizes are important in much real data, there are increasingly many settings in application where cluster sizes are not that small and for which the asymptotic approximations and large sample results established in this article are relevant, e.g., educational studies with large numbers of students (units) grouped within schools (clusters) [38], medical studies with large groups (clusters) of patients (units) treated at different hospitals [39], and settings where the data for each cluster are recorded at relatively high temporal frequency [40].

We begin by stating our main consistency result.

**Theorem 1**. *Assume (S1)–(S4) and Conditions (C1)–(C4) in the supplementary material are satisfied. Then for the independent cluster GLMM and conditional on the random effects* ${\dot{b}}_{i}$, *it holds that*


$$\begin{array}{lll} {}[{\hat{V}}_{\dot{b},y}\{b(\hat{\psi })-{\dot{b}}_{i}\}-V_{(\dot{b},y)|{\dot{b}}_{i}}({\hat{b}}_{i}-{\dot{b}}_{i})]∕V_{(\dot{b},y)|{\dot{b}}_{i}}({\hat{b}}_{i}-{\dot{b}}_{i})\overset{P}{\to }0, & \; & \end{array}$$

*for all* *i* = 1 , … , *m*, *where* ${\hat{V}}_{\dot{b},y}\{b(\hat{\psi })-{\dot{b}}_{i}\}$ *is defined in Eq (6).*

The above results states that for any cluster, the glmmTMB estimator, and thus the estimators of the CMSEP and UMSEP derived in Sect 2.2, are consistent for the *conditional* variance of the prediction gap given the random effects of that cluster. Importantly, this is not the unconditional variance the glmmTMB estimator sets out to estimate, nor is it the UMSEP or the CMSEP. This consistency result is also distinct from the results in [25], which are derived under an unconditional sampling framework. Theorem 1 explains why we observe differing prediction interval lengths for each cluster in Fig 1: even when clusters are exchangeable in the motivating Poisson GLMM example, the true conditional variances $V_{(\dot{b},y)|{\dot{b}}_{i}}$ differ for each cluster as they depend upon the corresponding random effect ${\dot{b}}_{i}$. Hence the glmmTMB estimator, which is consistent for this quantity, will also produce different prediction intervals across the clusters.

**Remark 4**. *Theorem 1 is based on conditioning on a finite subset of the random effects, which differs from conditioning on (a finite subset of) the observed* *y* *as in [14] and [12]. Put another way, Theorem 1 does not state whether the estimator for CMSEP in Sect 2.2.1, which so happens to take the same form as the* glmmTMB *estimator, is a consistent estimator for the true CMSEP.*

With regard to the true unconditional variance and true UMSEP, we provide the following remark.

**Remark 5**. *The* glmmTMB*estimator cannot, in general, be a consistent estimator of the unconditional variance of the prediction gap,* $V_{\dot{b},y}\{b(\hat{\psi })-\dot{b}\}$. *Similarly, the* glmmTMB*estimator cannot be a consistent estimator of the UMSEP,* $E_{\dot{b},y}[\{b(\hat{\psi })-\dot{b}\}{\left\{b(\hat{\psi })-\dot{b}\right\}}^{⊤}]$.

The above lack of consistency occurs due to employing the central approximation for $T_{1}$ (see Remark 2), as ${\left[-\partial_{bb^{⊤}}l\{b(\hat{\psi }),\hat{\psi }\}\right]}^{-1}={\left(Z_{i}^{⊤}{\hat{W}}_{i}Z_{i}\right)}^{-1}$ converges in probability to ${\left(Z_{i}^{⊤}{\dot{W}}_{i}Z_{i}\right)}^{-1}$, which is unconditionally a random variable and a function of the random effects ${\dot{b}}_{i}$. By contrast, the correct unconditional variance is actually $E_{\dot{b},y}\{{\left(Z_{i}^{⊤}{\dot{W}}_{i}Z_{i}\right)}^{-1}\}$, which is not a function of ${\dot{b}}_{i}$. We illustrate the differences between these via a concrete example in Sect 3.1.2.

Next, we offer a distributional result for the prediction gap of the PQL estimator.

**Theorem 2**. *Assume (S1)–(S4) and Conditions (C1)–(C4) are satisfied. Then for the independent cluster GLMM and conditional on the random effects* ${\dot{b}}_{i}$, *we have the following for each* *i* = 1 , … , *m*:

(a) *If* $mn_{i}^{-1}\to \infty$, *then* $n_{i}^{1∕2}({\hat{b}}_{i}-{\dot{b}}_{i})\overset{D}{\to }N(0,{\dot{K}}_{i})$.
(b) *If* $mn_{i}^{-1}\to \gamma \in (0,\infty )$, *then* $n_{i}^{1∕2}({\hat{b}}_{i}-{\dot{b}}_{i})\overset{D}{\to }N(0,{\dot{K}}_{i}+\gamma^{-1}\dot{G})$.
(c) *If* $mn_{i}^{-1}\to 0$, *then* $m^{1∕2}({\hat{b}}_{i}-{\dot{b}}_{i})\overset{D}{\to }N(0,\dot{G})$.

Theorem 2 can be summarized by stating that a correct finite sample approximation for the prediction gap ${\hat{b}}_{i}-{\dot{b}}_{i}$ when conditioning on ${\dot{b}}_{i}$, is given by $N(0,n_{i}^{-1}{\dot{K}}_{i}+m^{-1}\dot{G})$ distribution. Note Theorem 2c is the only part of the result where the asymptotic distribution does not involve the random effects $b_{i}$. An immediate consequence of this is that the case of $nn_{L}^{-1}\to 0$ offers one setting where the glmmTMB estimator can support an unconditional interpretation, and is in fact consistent for the unconditional variance of the prediction gap. Again, this asymptotic distributional result is distinct from the result for the prediction gap obtained in [25], which was derived in an unconditional framework and involves a normal scale-mixture distribution.

By combining Theorems 1 and 2, we obtain the following result which resolves the normality paradox.

**Corollary 1**. *Assume (S1)–(S4) and Conditions (C1)–(C4) are satisfied. Then normal prediction intervals constructed using the* glmmTMB *estimator asymptotically achieve nominal coverage.*

Theorems 1 and 2 are conditional on ${\dot{b}}_{i}$; however, Corollary 1 uses the fact that correct conditional coverage implies correct unconditional coverage to arrive at an unconditional result. Moreover, taken together the results above imply that one appropriate way to interpret prediction intervals constructed using the glmmTMB estimator plus a normal approximation is to do so conditionally on (a finite subset of) the random effects. By the equivalence of the glmmTMB estimator and the CMSEP and UMSEP estimators, the same interpretation can also be applied to predictions intervals constructed using the estimators of the CMSEP and UMSEP derived in Sect 2.2. Note this differs from the unconditional interpretation that is appropriate for prediction intervals based on the results in [25]; this is expected since the two intervals different in behavior substantially as exemplified from Fig 1.

#### 3.1.2 Poisson intercept-only GLMM example

To offer greater insight for the technical results above, we present a simple but insightful example involving a Poisson intercept-only GLMM. Consider the model f(yij|ḃi)=(yij!)−1 exp ⁡ (yijη˙ij−μ˙ij), where the mean is modeled as $\ln({\dot{\mu }}_{ij})={\dot{\eta }}_{ij}=\dot{\beta }+{ḃ}_{i}$, and ${ḃ}_{i}\overset{i.i.d.}{∼}N(0,{\dot{\sigma }}_{b}^{2})$. By condition (S4), we assume the variance component ${\dot{\sigma }}_{b}^{2}$ is known and $n_{i}=n$ for all *i* = 1 , … , *m* i.e., the design is balanced. Using PQL estimation to fit the GLMM, and following the developments of [25], for $nm^{-2}\to 0$ we can show the prediction gap for the *i*th cluster can be written as


b^i−ḃi=n−1 ∑j=1n{yij exp ⁡ (−β˙−ḃi)−1}+m−1 ∑i=1mḃi+op(b^i−ḃi). (7)

Suppose $mn^{-1}\to \gamma \in (0,\infty )$. Then conditional on ${ḃ}_{i}$, the asymptotic variance of $n^{1∕2}({\hat{b}}_{i}-{ḃ}_{i})$ is straightforwardly seen to be $\exp(-\dot{\beta }-{ḃ}_{i})+\gamma^{-1}{\dot{\sigma }}_{b}^{2}$, which agrees with Theorem 2b. When $mn^{-1}\to \infty$, the second term disappears and the asymptotic variance becomes $\exp(-\dot{\beta }-{ḃ}_{i})$ as in Theorem 2a. Finally, when $mn^{-1}\to 0$ case the first term disappears and the asymptotic variance of $m^{1∕2}({\hat{b}}_{i}-{ḃ}_{i})$ is ${\dot{\sigma }}_{b}^{2}$ as in Theorem 2c. Overall, from (7) an appropriate finite sample approximation of the conditional variance of ${\hat{b}}_{i}-{ḃ}_{i}$ can be given by n−1 exp ⁡ (−β˙−ḃi)+m−1σ˙b2.

Meanwhile, using the derivations from Sect 2.2.3, we can show the glmmTMB estimator for the uncertainty of ${\hat{b}}_{i}-{ḃ}_{i}$ is given by n−1 exp ⁡ (−β^−b^i)+m−1σ˙b2+op{min ⁡ (n−1,m−1)}. Since $\hat{\beta }$ and ${\hat{b}}_{i}$ converge in probability to $\dot{\beta }$ and ${ḃ}_{i}$, respectively, then we can further write the glmmTMB estimator as n−1 exp ⁡ (−β˙−ḃi)+m−1σ˙b2+op{min ⁡ (n−1,m−1)}. Ignoring the smaller order term, we see this leads to the same expression as the finite sample approximation of the conditional variance of ${\hat{b}}_{i}-{ḃ}_{i}$ based on (7). Thus when $nm^{-2}\to 0$, it follows the glmmTMB estimator is consistent for the conditional variance of ${\hat{b}}_{i}-{ḃ}_{i}$, irrespective of the precise rate of $mn^{-1}$. This agrees with Theorem 1.

In the case $mn^{-1}\to 0$, ${\hat{b}}_{i}\;-\;{ḃ}_{i}$ and ${ḃ}_{i}$ are asymptotically independent because $m^{-1∕2}\sum_{j=1}^{m}{ḃ}_{j}$ has the same $N(0,{\dot{\sigma }}_{b}^{2})$ distribution regardless of whether a finite subset of *ḃ* is constant or not. The glmmTMB, CMSEP and UMSEP estimators also reduce to the correct unconditional variance $m^{-1}{\dot{\sigma }}_{b}^{2}$, which is identical to the unconditional result of [25]. Therefore in this particular setting there is no difference between ${ḃ}_{i}$-conditional and unconditional inference. This is in agreement with Theorem 2c.

Consider now deriving an (unnormalized) asymptotic unconditional variance of ${\hat{b}}_{i}\;-\;{ḃ}_{i}$. This can be explicitly calculated from (7) as


Vb˙,y [n−1 ∑j=1n{yij exp ⁡ (−β˙−ḃi)−1}+m−1 ∑i=1mḃi]=EḃiVyi|ḃi [n−1 ∑j=1n{yij exp ⁡ (−β˙−ḃi)−1}+m−1 ∑i=1mḃi]+VḃiEyi|ḃi [n−1 ∑j=1n{yij exp ⁡ (−β˙−ḃi)−1}+m−1 ∑i=1mḃi]=n−1E ḃi{exp ⁡ (−β˙−ḃi)}+m−1σ˙b2=n−1 exp ⁡ (−β˙)exp ⁡ (0.5σ˙b2)+m−1σ˙b2.

This variance is evidently not n−1 exp ⁡ (−β˙−ḃi)+m−1σ˙b2, i.e., it is not the glmmTMB estimator and by extension not the estimators of UMSEP and CMSEP. Indeed, the glmmTMB estimator is unconditionally a random variable because of the use of a central approximation is used (see Remark 2). Moreover, the dependence on the estimated value of ${ḃ}_{i}$ is the reason why prediction intervals constructed using glmmTMB will differ in length between clusters.

[25] showed the sum of uncorrelated, (unconditionally) dependent random variables P=n−1∕2∑ ⁡j=1n{yij exp ⁡ (−β˙−ḃi)−1} converges in distribution not to a normal but in fact a normal scale-mixture distribution $\text{mixN}\{0,\exp(-\dot{\beta }-{ḃ}_{i}),N(0,{\dot{\sigma }}_{b}^{2})\}$. The latter is characterized by *P* having a $N\{0,\exp(-\dot{\beta }-{ḃ}_{i})\}$ distribution conditional on ${ḃ}_{i}$, and ${ḃ}_{i}∼N(0,{\dot{\sigma }}_{b}^{2})$. Let *H* ( ⋅ )  denote the cumulative distribution function of this normal scale-mixture distribution.

On the other hand, if we conditional on ${ḃ}_{i}$, then by the model definition $n^{-1∕2}P$ is a sum of i.i.d. random variables, and is independent of $m^{-1}\sum_{i=1}^{m}{ḃ}_{i}$. Thus the central limit theorem applies, and we have an asymptotic normality result for the (appropriately normalized) prediction gap ${\hat{b}}_{i}-{ḃ}_{i}$. This agrees with our result in Theorem 2, and together with the consistency of ${\hat{V}}_{\dot{b},y}\{b(\hat{\psi })\;-\;{\dot{b}}_{i}\}$ established in 2 previously explains the correct unconditional coverage as illustrated in Corollary 1.

To better elucidate the differences between inference conditional versus unconditional on ${ḃ}_{i}$ for the Poisson intercept-only GLMM, we now focus on the $mn^{-1}\to \infty$ case, i.e., when $n^{-1∕2}P$ is the dominating term in (7). First, conditional on ${ḃ}_{i}$, the quantity *P* converges to a normal distribution with mean zero and variance $\exp(-\dot{\beta }-{ḃ}_{i})$. As such, at the 1 − *α%* nominal level, the average length (normalized by *n*) of the conditional intervals is given by $2\Phi^{-1}(1\;-\;\alpha ∕2)E[\exp\{-0.5(\dot{\beta }+{ḃ}_{i})\}]=2\Phi^{-1}(1\;-\;\alpha ∕2)\exp(-0.5\dot{\beta })\exp(0.125{\dot{\sigma }}_{b}^{2})$. On the other hand, unconditional on ${ḃ}_{i}$, the length of the asymptotic central prediction interval is given by $H^{-1}(1-\alpha ∕2)-H^{-1}(\alpha ∕2)=2H^{-1}(1-\alpha ∕2)$, by the symmetry of the normal scale-mixture distribution. One can show then that, for small/large values of *α* and due to the heavy-tailedness of the normal scale-mixture distribution relative to the normal distribution, the unconditional interval will be larger/smaller than the average length of the conditional intervals.

**Fig 2:**
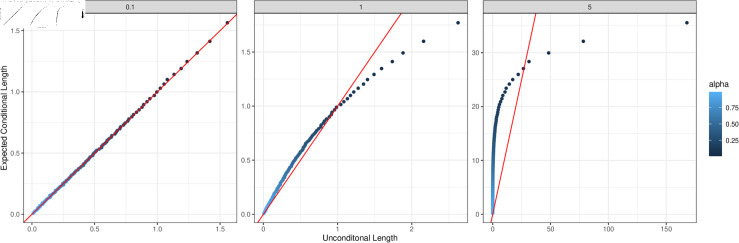
Expected conditional interval lengths versus unconditional interval lengths, for ${\dot{\sigma }}_{b}\in \{0.1,1,5\}$.

The discrepancy between the two interval lengths also depends on the value of ${\dot{\sigma }}_{b}^{2}$, and Fig 2 explores this in more detail. For small values of ${\dot{\sigma }}_{b}^{2}$, the expected lengths of the conditional intervals are roughly equal to the lengths of their unconditional counterparts across all significance levels. This is not surprising since, when ${\dot{\sigma }}_{b}^{2}$ is small, the normal scale-mixture distribution is close to a normal distribution. Indeed, the two intervals are identical when ${\dot{\sigma }}_{b}^{2}=0$. Conversely, when ${\dot{\sigma }}_{b}^{2}$ is large, the expected length of the conditional intervals is greater/smaller than the length of the unconditional intervals for higher/lower significance levels.

**Fig 3:**
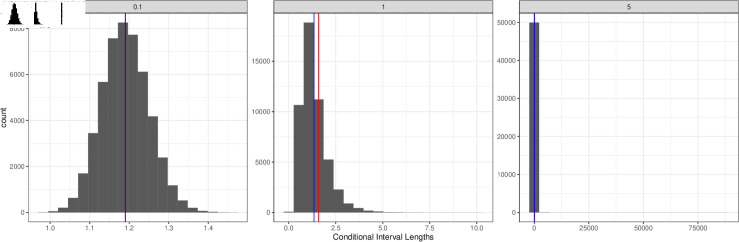
Histograms of conditional interval lengths, for a fixed significance level of 5*%* and ${\dot{\sigma }}_{b}\in \{0.1,1,5\}$. Blue line is the expected conditional interval length, and red line represents the corresponding unconditional interval length.

Finally, for a fixed 5% nominal level, Fig 3 examines the empirical distribution of 50,000 simulated conditional interval lengths, compared with the corresponding unconditional interval length. Results show larger values of ${\dot{\sigma }}_{b}^{2}$ result in the expected length of the conditional intervals being smaller than their unconditional counterpart. The variance of the conditional intervals’ length increases with increasing ${\dot{\sigma }}_{b}^{2}$, as the empirical distribution of lengths becomes increasingly right-skewed.

Ultimately, this Poisson intercept-only GLMM example can serve as a litmus test for practitioners to understand the consequences of performing analysis conditional on (a subset of) the random effects or not. Specifically, conditional inference implies prediction intervals will differ in length across each cluster, while unconditional inference implies prediction intervals will be of the same length. This may influence a practitioner’s decision on which inferential framework to work under.

### 3.2 Simulation study

We performed a simulation study to empirically verify the differences between the glmmTMB estimator and the results presented in [25]. Note [25] derived results for the PQL estimator, but for the purposes of this study we applied them directly to the Laplace estimator. The PQL and Laplace estimators are very similar when cluster sizes are sufficiently large (see Sect C of the supplementary material).

We simulated data from an independent cluster GLMM with $p_{f}=p_{r}=2$ fixed and random effect covariates as follows. First, we generated the fixed effect covariates $x_{ij}$ by setting the first element equal to one for a fixed intercept, and simulating the remaining elements from a standard normal distribution. As in assumption (S1), we then set the random effect covariates $z_{ij}=x_{ij}$. Next, we set the 2-vector of true fixed effect coefficients as $\dot{\beta }={\left(-0.1,0.1\right)}^{⊤}$, the 2 × 2 random effects covariance matrix $\dot{G}=I_{2}$, and simulated the random effects as ${\dot{b}}_{i}\overset{i.i.d.}{∼}N(0,\dot{G})$. Finally, conditional on ${\dot{b}}_{i}$ the responses $y_{ij}$ were generated from a Poisson distribution with log link i.e., $\ln(\mu_{ij})=x_{ij}^{⊤}\dot{\beta }\;+\;z_{ij}^{⊤}{\dot{b}}_{i}$. We varied the number of clusters as *m* = { 25 , 50 , 100 , 200 }  and the cluster sizes $n_{i}=n=\{25,50,100,200\}$. For each combination of  ( *m* , *n* ) , we simulated 1000 datasets.

For each simulated dataset, we fitted the corresponding GLMM using glmmTMB with $\dot{G}$ set as known. We then examined the unconditional empirical coverage probability of 95% prediction intervals constructed from the glmmTMB estimator (as derived in Sect 2.2.3), and compared these to prediction intervals constructed using the results in [25] as well as from lme4. Note to construct the unconditional intervals of [25], we compute the relevant asymptotic variance and the quantiles of the relevant normal scale-mixture distribution via direct simulation with 10000 samples. For simplicity, we focus on the prediction intervals for the random effects of the first cluster ${\dot{b}}_{1}$, and for quantities ${\dot{\beta }}_{1}\;+\;{ḃ}_{11}$ and ${\dot{\beta }}_{2}+{ḃ}_{12}$.

For the random effect predictions, the empirical coverage probabilities of the glmmTMB and unconditional intervals of [25] were fairly close to the nominal level across all combinations of  ( *m* , *n* ) , with the exception of a tendency to overcover when *n* = 25 (Table 1). This is concordant with our theorems in Sect 3.1 and Corollary 1. By contrast, the prediction intervals from lme4 undercover severely when *n* grows at a rate faster than or equal to *m*, in agreement with the discussion in Sect 2.2.4. Turning to prediction interval widths, the conditional intervals from glmmTMB had a smaller average length than the unconditional intervals of [25] (Table 2). However, the unconditional intervals also have higher coverage in this case. On the other hand, Table 3 presents the same results but scaled by *n*, from which we see the variance of the interval lengths using the glmmTMB and lme4 estimators remain roughly constant as  ( *m* , *n* )  grow. This implies the standard errors are converging to a random variable rather than a constant.

**Table 1:** Empirical coverage probabilities of prediction intervals for ${\dot{b}}_{1}$, constructed using glmmTMB, lme4, and the unconditional approach of [25].

	*m*	Random Intercept				Random Slope			
		*n* = 25	*n* = 50	*n* = 100	*n* = 200	*n* = 25	*n* = 50	*n* = 100	*n* = 200
Unconditonal	25	0.956	0.961	0.961	0.958	0.956	0.943	0.948	0.958
	50	0.964	0.954	0.954	0.954	0.963	0.956	0.947	0.954
	100	0.963	0.963	0.949	0.955	0.957	0.962	0.961	0.958
	200	0.962	0.955	0.956	0.950	0.968	0.956	0.954	0.951
glmmTMB	25	0.949	0.953	0.953	0.955	0.945	0.939	0.948	0.955
	50	0.932	0.941	0.953	0.950	0.945	0.946	0.952	0.950
	100	0.952	0.949	0.942	0.958	0.929	0.945	0.950	0.955
	200	0.941	0.952	0.955	0.938	0.964	0.955	0.946	0.950
lme4	25	0.819	0.744	0.636	0.518	0.693	0.614	0.533	0.408
	50	0.847	0.810	0.719	0.623	0.783	0.737	0.615	0.499
	100	0.919	0.852	0.809	0.728	0.837	0.811	0.752	0.622
	200	0.913	0.905	0.870	0.805	0.908	0.867	0.798	0.713

**Table 2:** Empirical average interval lengths of prediction intervals for ${\dot{b}}_{1}$, constructed using glmmTMB, lme4, and the unconditional approach of [25].

	*m*	Random Intercept				Random Slope			
		*n* = 25	*n* = 50	*n* = 100	*n* = 200	*n* = 25	*n* = 50	*n* = 100	*n* = 200
Unconditional	25	1.392	1.121	0.969	0.890	1.192	1.004	0.899	0.824
	50	1.277	0.977	0.783	0.687	1.082	0.824	0.699	0.633
	100	1.256	0.903	0.690	0.554	1.010	0.734	0.592	0.479
	200	1.243	0.866	0.642	0.480	0.981	0.689	0.527	0.398
glmmTMB	25	1.217	1.036	0.931	0.865	1.076	0.949	0.878	0.832
	50	1.077	0.880	0.749	0.662	0.910	0.779	0.682	0.618
	100	1.016	0.762	0.624	0.525	0.818	0.647	0.545	0.472
	200	0.943	0.726	0.559	0.441	0.752	0.583	0.471	0.377
lme4	25	0.902	0.645	0.471	0.338	0.680	0.489	0.360	0.247
	50	0.901	0.660	0.477	0.336	0.678	0.513	0.364	0.245
	100	0.924	0.636	0.467	0.329	0.690	0.488	0.355	0.238
	200	0.894	0.660	0.473	0.329	0.681	0.494	0.364	0.237

**Table 3:** Scaled empirical interval length variance of prediction intervals for ${\dot{b}}_{1}$, separately constructed using glmmTMB and lme4.

	*m*	Random Intercept				Random Slope			
		*n* = 25	*n* = 50	*n* = 100	*n* = 200	*n* = 25	*n* = 50	*n* = 100	*n* = 200
glmmTMB	25	0.083	0.080	0.072	0.056	0.066	0.054	0.036	0.024
	50	0.107	0.108	0.112	0.088	0.087	0.074	0.068	0.049
	100	0.120	0.132	0.132	0.104	0.098	0.096	0.076	0.059
	200	0.126	0.170	0.156	0.112	0.117	0.122	0.104	0.072
lme4	25	0.165	0.196	0.240	0.264	0.153	0.160	0.160	0.176
	50	0.160	0.188	0.244	0.244	0.147	0.156	0.180	0.160
	100	0.150	0.184	0.212	0.216	0.138	0.152	0.152	0.160
	200	0.143	0.202	0.204	0.208	0.142	0.160	0.160	0.144

## 4 Discussion

In this article, we examined several well-known measures of prediction uncertainty for random effects’ predictions in GLMMs, namely estimators of CMSEP and UMSEP, as well as the estimators used in the software packages glmmTMB and lme4. We demonstrated the first three measures arrive at the same estimator despite having different aims, while the lme4 estimator differs from the other three. When the fixed and random effects are partnered, the lme4 estimator results in severe undercoverage of the true random effects when the cluster size is growing faster than the number of clusters. We also leveraged the theoretical results of [25] to explain why using the glmmTMB estimator and a normal assumption yields asymptotically correct inference for the true random effects. Our derivations showed that this glmmTMB procedure can be asymptotically justified by conditioning on the random effects ${\dot{b}}_{i}$ for a finite number of clusters; this is contrary to the underlying original goal of glmmTMB and UMSEP, which is to make unconditional inference. Through a motivating example and simulation studies, we empirically demonstrate the differences between the conditional inference provided by typical software packages, and correct unconditional inference. Moreover, the litmus test as given by the Poisson intercept-only GLMM (based on the exchangeablility of clusters) leads to prediction intervals of the same length for the random intercepts of any cluster when working in an unconditional framework. On the other hand, inference conditional on ${\dot{b}}_{i}$ allows prediction interval lengths to differ for each cluster.

It is important to note the theoretical developments in this article suggest that an order of magnitude difference between *m* and *n* is unlikely to greatly deteriorate the asymptotic approximations which lme4 and glmmTMB essentially use, so long as the *actual values* of the cluster sizes themselves (regardless of the ratio to the number of clusters) are sufficiently large for the asymptotic approximations to be accurate. That is, the findings of this work can still be relevant in practical applications where the number of clusters greatly exceed the cluster size, provided the actual values of the latter are not extremely small. This is based on our theory requiring $mn^{-2}\to 0$ i.e., the number of clusters are not growing faster than the cluster size squared, and indeed our numerical results support this, e.g., coverage of the intervals are close to nominal when *m* = 200 and *n* = 25. This is also consistent with recent simulation results in, e.g., [20,41], who consider even smaller sample cluster sizes of *n* = 10. Our theory and simulation results further suggest the coverage of lme4 random effects prediction intervals tend toward nominal (from undercoverage) even in the partnered case, as the ratio of *m* ∕ *n* increases.

Although our conclusions hold under the assumptions made in [25], they offer a first step towards offering justification for procedures in prediction inference in GLMMs previously lacking in the literature. We conjecture some of the assumptions can be relaxed without affecting the results greatly, and establishing this formally is an interesting future direction to pursue. Furthermore, the viability - both in practice and theory - of inference conditional on *y* or $y_{i}$ is not explored deeply in this article, and is another avenue of further research to pursue. Finally, beyond Remark 1 for the CMSEP, it is important to acknowledge resampling approaches such as the parametric bootstrap and variations thereof are another class of approaches used to construct prediction intervals in GLMMs; see for instance [42,43] as well as [44,45] on the use of the jackknife for small area estimation. We conjecture the way in which resampling is performed e.g., conditional on the random effects or not, is likely to affect the properties of the resulting prediction intervals, and may be closely related to the theory and conclusions reached in this article.

Finally, GLMMs are commonly used in situations where there exist missing data [46,47], and so the extension of our theory and finite sample results to incorporate various missing data patterns is an important avenue of future research. As a starting point, although our theoretical framework is currently not set up to incorporate any missingness in the response or fixed/random effect covariates, the fact that we allow uneven cluster sizes suggests we could rely on standard literature in the case of Missing Completely at Random (MCAR) and perform complete case analysis without any theoretical properties changing [47]. On the other hand, we conjecture there are likely to be biases in both point and interval estimates if our major theoretical results are directly applied to the case of (Missing Not at Random) MNAR responses and/or covariates, and this would also be consistent with standard MNAR literature (e.g., [46,48]). Finally, in the case of (Missing at Random) MAR data, a common approach is to employ some form of multiple imputation [49]. We conjecture our asymptotic developments which condition on $b_{i}$ e.g., Theorems 1 and 2, will still be valid for fixed effects (which the $b_{i}$ are in this case) inference if classic methods of imputation such as using Rubin’s rules and increasing the variance of the asymptotic approximating normal distribution are employed [50]. However, further investigation is required when it comes to random effects inference/prediction intervals in the unconditional case, since the extra variability from imputation would need to be incorporated into our theoretical results, either within the normal mixture distribution component or the normal distribution component of the asymptotic approximating distribution.

## Supporting information

S1 Fig 1 Comparison of glmmTMB and lme4 point predictions of the random effects. (TIF)

S2 Fig 2Naive interval lengths versus unconditional interval lengths, for ${\dot{\sigma }}_{b}\in \{0.1,1,5\}$.(TIF)

S1 Appendix Proofs and extra results. (PDF)
