# Supplementary figures and images for: Inferential procedures for random effects in generalized linear mixed models
Source: PLoS One. 2025 Apr 16;20(4):e0320797. doi: 10.1371/journal.pone.0320797 (PMC12002512; doi:10.1371/journal.pone.0320797)

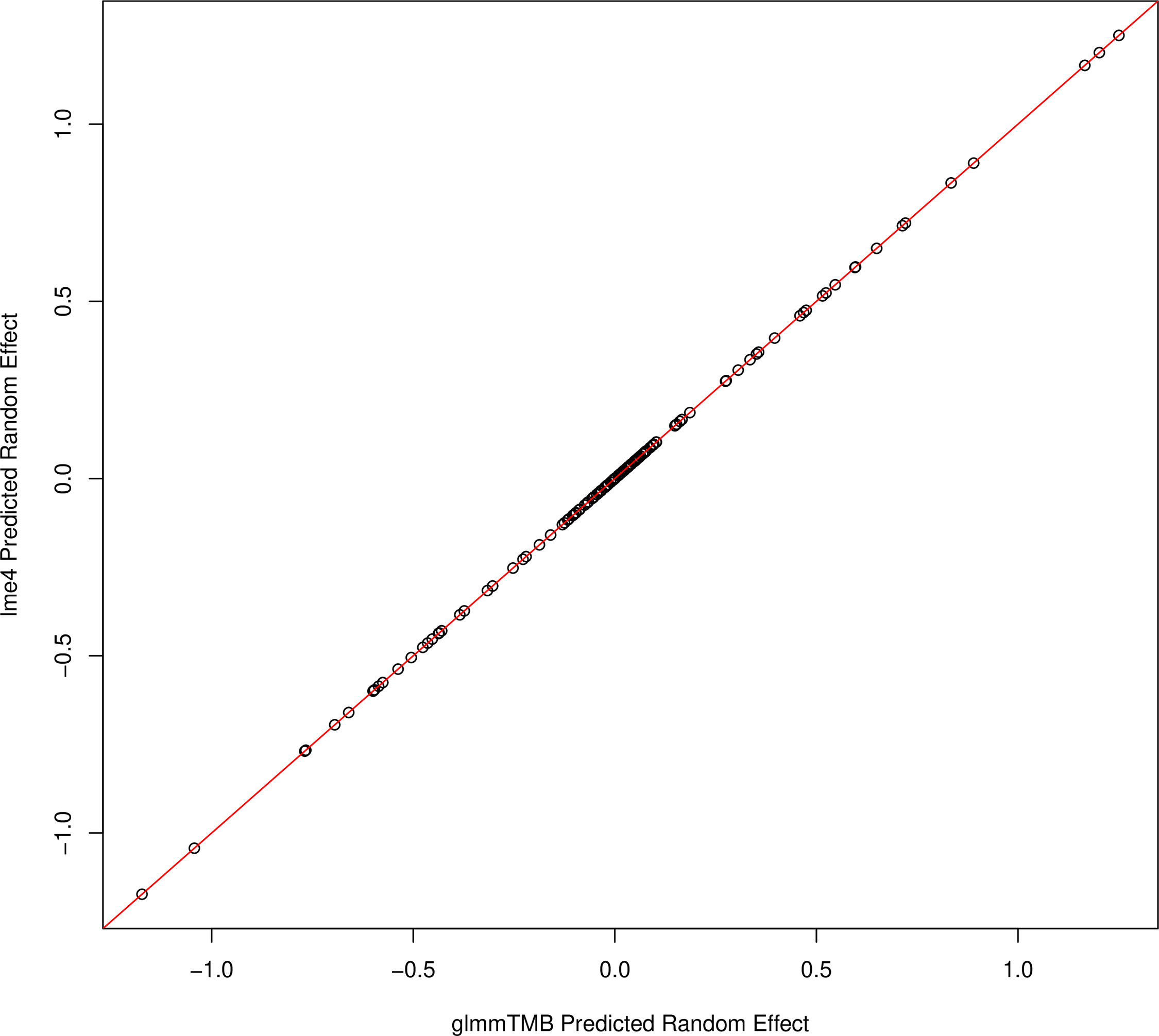

Supplement: S1 Fig 1 — (TIF) [file pone.0320797.s001.tif]

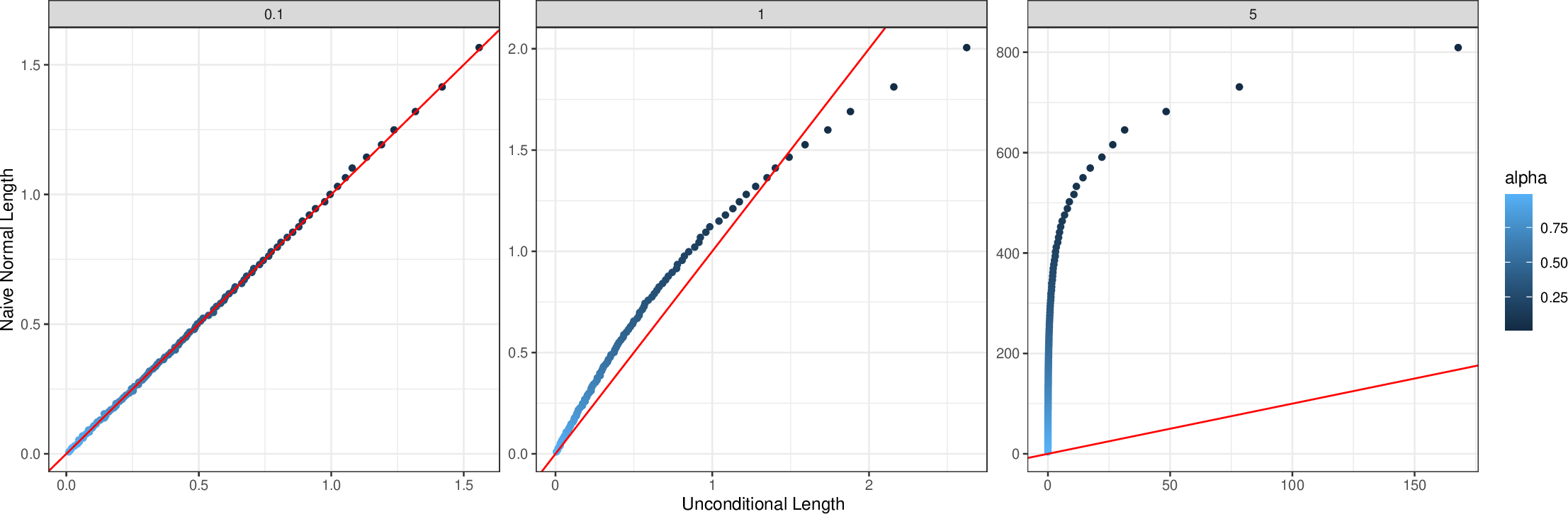

Supplement: S2 Fig 2 — (TIF) [file pone.0320797.s002.tif]
