# Supplementary material for: Inferential procedures for random effects in generalized linear mixed models
Source: PLoS One. 2025 Apr 16;20(4):e0320797. doi: 10.1371/journal.pone.0320797 (PMC12002512; doi:10.1371/journal.pone.0320797)
Supplement: S1 Appendix — (PDF) [file pone.0320797.s003.pdf]

Supplementary Material

A Proofs

For an arbitrary matrix $\boldsymbol{C}$, let $\boldsymbol{C}_{[i:j,k:l]}$ denote the sub-matrix comprising the ith to jth row and kth to lth column of $\boldsymbol{C}$ and $\boldsymbol{C}_{[i,]}$ and $\boldsymbol{C}_{[,j]}$ denote the ith row and jth column respectively. Similarly, for a vector $\boldsymbol{c}$ we let $\boldsymbol{c}_{[i:j]}$ denote the sub-vector formed by taking the ith to jth components; the quantity $\boldsymbol{c}_{[i]}$ simply denotes the ith component of $\boldsymbol{c}$. Lastly, for a rank three tensor $\mathfrak{C}$, let $\mathfrak{C}_{[i:j,k:l,n:m]}$ denote the sub-tensor comprising the ith to jth row, kth to lth column, and nth to mth surface of $\mathfrak{C}$. Analogous to the notation for matrices and vectors, we also let $\mathfrak{C}_{[i,,]}$ denote the matrix formed by extracting the ith row of $\mathfrak{C}$, and so on. Let $\boldsymbol{\mu}_i(\boldsymbol{\theta}) = \{a'(\eta_{i1}), \ldots, a'(\eta_{im})\}^\top$, $\boldsymbol{\mu}(\boldsymbol{\theta}) = \{\boldsymbol{\mu}_1^\top(\boldsymbol{\theta}), \ldots, \boldsymbol{\mu}_m(\boldsymbol{\theta})^\top\}^\top$, $\boldsymbol{W}_i = \dot{\phi}^{-1}\text{diag}\{a''(\eta_{i1}), \ldots, a''(\eta_{im})\}$, and $\boldsymbol{W} = \text{blockdiag}(\boldsymbol{W}_1, \ldots, \boldsymbol{W}_m)$. Furthermore, write $\dot{\mu}_{ij} = a''(\dot{\eta}_{ij})$, $\dot{\boldsymbol{\mu}}_i = \boldsymbol{\mu}_i(\dot{\boldsymbol{\theta}})$ and $\dot{\boldsymbol{\mu}} = \boldsymbol{\mu}(\dot{\boldsymbol{\theta}})$, and let $\otimes$ denote the Kronecker product operator, $\boldsymbol{I}_m$ denote the $m \times m$ identity matrix, and $\mathbf{1}_m$ denote a matrix or vector of ones, with dimension indicated by the relevant subscripts.

We require the following regularity conditions for the GLMM.

(C1) The function $a(\eta)$ is at least three times continuously differentiable in its domain, with $0 < v_0 \leq a''(\eta) \leq v_0^{-1} < \infty$ and $|a'''(\eta)| \leq v_0^{-1} < \infty$ for some sufficiently small constant v_0.

(C2) For every $i = 1, \ldots, m$ and $j = 1, \ldots, n_i$, there exists a sufficiently large constant V_1 such that $\|\boldsymbol{x}_{ij}\|_\infty < V_1$ where $\|\cdot\|_\infty$ is the maximum norm. Furthermore, for all $i = 1, \ldots, m$ the matrices $\lim_{n_i \to \infty}(n_i^{-1}\boldsymbol{X}_i^\top \dot{\boldsymbol{W}}_i \boldsymbol{X}_i)^{-1} = \dot{\boldsymbol{K}}_i$ and $\lim_{m,n_L \to \infty} m^{-1}\sum_{i=1}^m n n_i^{-1}(n_i^{-1}\boldsymbol{X}_i^\top \dot{\boldsymbol{W}}_i \boldsymbol{X}_i)^{-1} = \dot{\boldsymbol{K}}$ are positive definite with minimum and maximum eigenvalues bounded from above and below by v_1^{-1} and v_1 respectively, for a sufficiently small constant v_1.

(C3) The vector of true parameters $\dot{\boldsymbol{\theta}} = (\dot{\boldsymbol{\beta}}^\top, \dot{\boldsymbol{b}}^\top)^\top$, where $\dot{\boldsymbol{b}} = (\dot{\boldsymbol{b}}_1^\top, \ldots, \dot{\boldsymbol{b}}_m^\top)^\top$, is an interior point in some compact set $\Theta \subset \mathbb{R}^{(m+1)p}$.

(C4) For all $i = 1, \dots, m$ and $n_i \in \mathbb{N}$, it holds that $E([n_i^{1/2}(\boldsymbol{X}_i^\top \dot{\boldsymbol{W}}_i \boldsymbol{X}_i + \dot{\boldsymbol{G}}^{-1})^{-1}\{\boldsymbol{X}_i^\top(\boldsymbol{y}_i - \dot{\boldsymbol{\mu}}_i) - \dot{\boldsymbol{G}}^{-1}\dot{\boldsymbol{b}}_i\}]^4) < \infty$, where the power and expectation are applied component-wise.

The above conditions are similar to those required in proofs of asymptotic normality in various other settings, e.g., Fan and Peng, 2004; Lyu and Welsh, 2021. Conditions (C1) - (C3) are needed to guarantee the existence and regular behavior of the asymptotic variance for the PQL estimating function, and to establish a Lindeberg condition needed to obtain a central limit theorem. Condition (C4) is needed to bound the order of $\|\hat{\boldsymbol{\theta}} - \dot{\boldsymbol{\theta}}\|_\infty$, and is satisfied by many distributions e.g. Poisson and Binomial, when the random effects are normally distributed. Conditions on the fourth moment of the score functions (or variations thereof) are also fairly common, especially when the dimension is increasing, e.g., van de Geer and Müller, 2012.

Assume simplifying assumptions (S1) - (S4) and Conditions (C1) -(C4) are satisfied. Then from Ning et al. (2024) we have the key equation

$$\hat{\boldsymbol{b}}_i - \dot{\boldsymbol{b}}_i = -m^{-1}\sum_{i=1}^{m}\dot{\boldsymbol{b}}_i + (\boldsymbol{Z}_i^\top \dot{\boldsymbol{W}}_i \boldsymbol{Z}_i)^{-1}[\boldsymbol{Z}_i^\top\{\boldsymbol{y}_i - \boldsymbol{\mu}_i(\dot{\boldsymbol{\theta}})\}] + o_p(\hat{\boldsymbol{b}}_i - \dot{\boldsymbol{b}}_i) \tag{1}$$

for $i = 1, \dots, m$, and for $i = 1, \dots, m$. Ning et al. (2024) prove that $(\boldsymbol{Z}_i^\top \dot{\boldsymbol{W}}_i \boldsymbol{Z}_i)^{-1}[\boldsymbol{Z}_i^\top\{\boldsymbol{y}_i - \boldsymbol{\mu}_i(\dot{\boldsymbol{\theta}})\}]$ converges in distribution to the normal scale-mixture distribution.

We begin by proving a convergence result for $m\{-\partial_{\boldsymbol{\beta}\boldsymbol{\beta}^\top}\ell_{PQL}(\hat{\boldsymbol{\beta}})\}^{-1}$, which appears in the`glmmTMB` estimator, in (6).

Lemma 1. *Let $\ell_{PQL}(\boldsymbol{\beta})$ denote the PQL approximation to the marginal log-likelihood $l(\boldsymbol{\beta})$. Assume Conditions (C1) - (C4) are satisfied, and $mn_L^{-2} \to 0$. Then as $m, n_L \to \infty$, and conditional on the random effects $\dot{\boldsymbol{b}}_i$, it holds that $m\{-\partial_{\boldsymbol{\beta}\boldsymbol{\beta}^\top}\ell_{PQL}(\hat{\boldsymbol{\beta}})\}^{-1} = \dot{\boldsymbol{G}} + O_p(n_L^{-1})$.*

Proof. We begin by deriving an expression for $\partial_{\boldsymbol{\beta}^\top}\boldsymbol{b}(\boldsymbol{\beta})$. With the true random effects covariance matrix $\dot{\boldsymbol{G}}$ and true dispersion parameter $\dot{\phi}$ assumed to be known, then we have

$$\begin{aligned}
-n[\partial_{\boldsymbol{b}\boldsymbol{b}^\top} l\{\boldsymbol{b}(\boldsymbol{\beta}), \boldsymbol{\beta}\}]^{-1} &= n(\boldsymbol{Z}^\top \tilde{\boldsymbol{W}} \boldsymbol{Z} + \boldsymbol{I}_m \otimes \dot{\boldsymbol{G}}^{-1})^{-1} \\
&= n(\boldsymbol{Z}^\top \tilde{\boldsymbol{W}} \boldsymbol{Z})^{-1} - n(\boldsymbol{Z}^\top \tilde{\boldsymbol{W}} \boldsymbol{Z})^{-1}(\boldsymbol{I}_m \otimes \dot{\boldsymbol{G}}^{-1})(\boldsymbol{Z}^\top \tilde{\boldsymbol{W}} \boldsymbol{Z} + \boldsymbol{I}_m \otimes \dot{\boldsymbol{G}}^{-1})^{-1} \\
&= n(\boldsymbol{Z}^\top \tilde{\boldsymbol{W}} \boldsymbol{Z})^{-1} + O_p(n_L^{-1}),
\end{aligned}$$

where the second line follows by the Woodbury matrix identity and $\tilde{\boldsymbol{W}}$ is $\boldsymbol{W}$ with $\boldsymbol{\theta}$ replaced

with $\tilde{\boldsymbol{\theta}} = \{\boldsymbol{\beta}, \boldsymbol{b}(\boldsymbol{\beta})\}$. Using $\boldsymbol{Z}_i = \boldsymbol{X}_i$, we also have $\partial_{\boldsymbol{b}\boldsymbol{\beta}^\top} l\{\boldsymbol{b}(\boldsymbol{\beta}), \boldsymbol{\beta}\} = -\boldsymbol{Z}^\top \tilde{\boldsymbol{W}} \boldsymbol{Z}(\mathbf{1}_m \otimes \boldsymbol{I}_p)$. Thus

$$\begin{aligned}\partial_{\boldsymbol{\beta}^\top} \boldsymbol{b}(\boldsymbol{\beta}) &= -[\partial_{\boldsymbol{b}\boldsymbol{b}^\top} l\{\boldsymbol{b}(\boldsymbol{\beta}), \boldsymbol{\beta}\}]^{-1} \partial_{\boldsymbol{b}\boldsymbol{\beta}^\top} l\{\boldsymbol{b}(\boldsymbol{\beta}), \boldsymbol{\beta}\} \\ &= -\mathbf{1}_m \otimes \boldsymbol{I}_p + \boldsymbol{\Gamma},\end{aligned} \tag{2}$$

where $\boldsymbol{\Gamma}$ is a component-wise $O_p(n^{-1})$ $mp \times p$ matrix given by

$$\begin{aligned}\boldsymbol{\Gamma} &= (\boldsymbol{Z}^\top \tilde{\boldsymbol{W}} \boldsymbol{Z})^{-1} (\boldsymbol{I}_m \otimes \dot{\boldsymbol{G}}^{-1}) (\boldsymbol{Z}^\top \tilde{\boldsymbol{W}} \boldsymbol{Z} + \boldsymbol{I}_m \otimes \dot{\boldsymbol{G}}^{-1})^{-1} \boldsymbol{Z}^\top \tilde{\boldsymbol{W}} \boldsymbol{Z} (\mathbf{1}_m \otimes \boldsymbol{I}_p) \\ &= \begin{bmatrix} (\boldsymbol{X}_1^\top \tilde{\boldsymbol{W}}_1 \boldsymbol{X}_1)^{-1} \dot{\boldsymbol{G}}^{-1} (\boldsymbol{X}_1^\top \tilde{\boldsymbol{W}}_1 \boldsymbol{X}_1 + \dot{\boldsymbol{G}}^{-1})^{-1} \boldsymbol{X}_1^\top \tilde{\boldsymbol{W}}_1 \boldsymbol{X}_1 \\ \vdots \\ (\boldsymbol{X}_m^\top \tilde{\boldsymbol{W}}_m \boldsymbol{X}_m)^{-1} \dot{\boldsymbol{G}}^{-1} (\boldsymbol{X}_m^\top \tilde{\boldsymbol{W}}_m \boldsymbol{X}_m + \dot{\boldsymbol{G}}^{-1})^{-1} \boldsymbol{X}_m^\top \tilde{\boldsymbol{W}}_m \boldsymbol{X}_m \end{bmatrix}.\end{aligned}$$

This expression can be further simplified because

$$\begin{aligned}&(\boldsymbol{X}_i^\top \tilde{\boldsymbol{W}}_i \boldsymbol{X}_i)^{-1} \dot{\boldsymbol{G}}^{-1} (\boldsymbol{X}_i^\top \tilde{\boldsymbol{W}}_i \boldsymbol{X}_i + \dot{\boldsymbol{G}}^{-1})^{-1} \boldsymbol{X}_i^\top \tilde{\boldsymbol{W}}_i \boldsymbol{X}_i \\ &= (\dot{\boldsymbol{G}} \boldsymbol{X}_i^\top \tilde{\boldsymbol{W}}_i \boldsymbol{X}_i)^{-1} \{\boldsymbol{I}_p + (\dot{\boldsymbol{G}} \boldsymbol{X}_i^\top \tilde{\boldsymbol{W}}_i \boldsymbol{X}_i)^{-1}\}^{-1} \\ &= (\dot{\boldsymbol{G}} \boldsymbol{X}_i^\top \tilde{\boldsymbol{W}}_i \boldsymbol{X}_i)^{-1} [\dot{\boldsymbol{G}} \boldsymbol{X}_i^\top \tilde{\boldsymbol{W}}_i \boldsymbol{X}_i - \dot{\boldsymbol{G}} \boldsymbol{X}_i^\top \tilde{\boldsymbol{W}}_i \boldsymbol{X}_i \{\boldsymbol{I}_p + (\dot{\boldsymbol{G}} \boldsymbol{X}_i^\top \tilde{\boldsymbol{W}}_i \boldsymbol{X}_i)^{-1}\}^{-1}] \\ &= \boldsymbol{I}_p - \{\boldsymbol{I}_p + (\dot{\boldsymbol{G}} \boldsymbol{X}_i^\top \tilde{\boldsymbol{W}}_i \boldsymbol{X}_i)^{-1}\}^{-1} \\ &= (\boldsymbol{I}_p + \dot{\boldsymbol{G}} \boldsymbol{X}_i^\top \tilde{\boldsymbol{W}}_i \boldsymbol{X}_i)^{-1},\end{aligned}$$

for any $i = 1, \ldots, m$, where the third and last lines follow from the Woodbury matrix identity. As an aside, these derivations provide some backing for the approximation of U_2 by U_3 in the main text.

Remark 1. *Under the model considered in Ning et al. (2024),* $[\partial_{\boldsymbol{b}\boldsymbol{b}^\top} l\{\boldsymbol{b}(\dot{\boldsymbol{\beta}}), \dot{\boldsymbol{\beta}}\}]^{-1} \partial_{\boldsymbol{b}\boldsymbol{\beta}^\top} l\{\boldsymbol{b}(\dot{\boldsymbol{\beta}}), \dot{\boldsymbol{\beta}}\} = \mathbf{1}_m \otimes \boldsymbol{I}_p + o_p(1)$. *Thus, if the smaller order term is ignored, a further approximation to U_2 of the type employed in Kackar and Harville (1984) or Prasad and Rao (1990) is not required in this case. The resulting estimator is then the same as the estimator derived when we treat $\mathbf{b}(\boldsymbol{\psi})$ as if it is not a function of $\boldsymbol{y}$.*

Next, the PQL approximation to the log-likelihood is given by

$$\ell_{PQL}(\boldsymbol{\beta}) = \sum_{i=1}^{m}\sum_{j=1}^{n_i} \ln f\{y_{ij}|\boldsymbol{\beta}, \boldsymbol{b}_i(\boldsymbol{\beta})\} - \frac{1}{2}\sum_{i=1}^{m} \boldsymbol{b}_i(\boldsymbol{\beta})^\top \dot{\boldsymbol{G}}^{-1}\boldsymbol{b}_i(\boldsymbol{\beta}),$$

where $\boldsymbol{b}(\boldsymbol{\beta})$ satisfies $\partial_{\boldsymbol{b}} l\{\boldsymbol{b}(\boldsymbol{\beta}), \boldsymbol{\beta}\} = \boldsymbol{0}$. That is, $\boldsymbol{b}(\boldsymbol{\beta})$ is the maximiser of $l(\boldsymbol{b}, \boldsymbol{\beta})$ over $\boldsymbol{b}$ for a given $\boldsymbol{\beta}$, and thus an implicit function of $\boldsymbol{\beta}$. If we view $\ell_{PQL}(\boldsymbol{\beta})$ as a function of both $\boldsymbol{\beta}$ and $\boldsymbol{b}(\boldsymbol{\beta})$, and denote this by $\ell_{PQL}\{\boldsymbol{\beta}, \boldsymbol{b}(\boldsymbol{\beta})\}$, then applying the multivariate chain rule we obtain

$$\begin{aligned}
\nabla_{\boldsymbol{\beta}}\ell_{PQL}(\boldsymbol{\beta}) &= \partial_{\boldsymbol{\beta}}\ell_{PQL}\{\boldsymbol{\beta}, \boldsymbol{b}(\boldsymbol{\beta})\} + \partial_{\boldsymbol{\beta}}\boldsymbol{b}(\boldsymbol{\beta})^\top \partial_{\boldsymbol{b}(\boldsymbol{\beta})}\ell_{PQL}\{\boldsymbol{\beta}, \boldsymbol{b}(\boldsymbol{\beta})\} \\
&= \dot{\phi}^{-1}\boldsymbol{X}^\top(\boldsymbol{y} - \tilde{\boldsymbol{\mu}}) - (\boldsymbol{1}_m^\top \otimes \boldsymbol{I}_p + \boldsymbol{\Gamma}^\top)\{\dot{\phi}^{-1}\boldsymbol{Z}^\top(\boldsymbol{y} - \tilde{\boldsymbol{\mu}}) - (\boldsymbol{I}_m \otimes \dot{\boldsymbol{G}}^{-1})\boldsymbol{b}(\boldsymbol{\beta})\} \\
&= \sum_{i=1}^{m} \dot{\boldsymbol{G}}^{-1}\boldsymbol{b}_i(\boldsymbol{\beta}) + \boldsymbol{\Gamma}^\top\{\dot{\phi}^{-1}\boldsymbol{Z}^\top(\boldsymbol{y} - \tilde{\boldsymbol{\mu}}) - (\boldsymbol{I}_m \otimes \dot{\boldsymbol{G}}^{-1})\boldsymbol{b}(\boldsymbol{\beta})\},
\end{aligned}$$

where $\tilde{\boldsymbol{\mu}} = \boldsymbol{\mu}(\tilde{\boldsymbol{\theta}})$, since under the $\boldsymbol{X}_i = \boldsymbol{Z}_i$ assumption

$$(\boldsymbol{1}_m^\top \otimes \boldsymbol{I}_p)\boldsymbol{Z}^\top(\boldsymbol{y} - \tilde{\boldsymbol{\mu}}) = \boldsymbol{X}^\top(\boldsymbol{y} - \tilde{\boldsymbol{\mu}}).$$

Next, again by the multivariate chain rule, we obtain the total derivative of $\nabla_{\boldsymbol{\beta}}\ell_{PQL}(\boldsymbol{\beta})$ with respect to $\boldsymbol{\beta}$ as

$$\nabla_{\boldsymbol{\beta}\boldsymbol{\beta}^\top}\ell_{PQL}(\boldsymbol{\beta}) = \partial_{\boldsymbol{\beta}^\top}\nabla_{\boldsymbol{\beta}}\ell_{PQL}(\boldsymbol{\beta}) + \{\partial_{\boldsymbol{b}(\boldsymbol{\beta})}^\top \nabla_{\boldsymbol{\beta}}\ell_{PQL}(\boldsymbol{\beta})\}\partial_{\boldsymbol{\beta}^\top}\boldsymbol{b}(\boldsymbol{\beta}).$$

We have

$$\begin{aligned}
&\partial_{\boldsymbol{\beta}^\top}\left\{\sum_{i=1}^{m} \dot{\boldsymbol{G}}^{-1}\boldsymbol{b}_i(\boldsymbol{\beta})\right\} = \boldsymbol{0}_{p\times p} \\
&\partial_{\boldsymbol{b}(\boldsymbol{\beta})^\top}\left\{\sum_{i=1}^{m} \dot{\boldsymbol{G}}^{-1}\boldsymbol{b}_i(\boldsymbol{\beta})\right\} = \boldsymbol{1}_{mp}^\top \otimes \dot{\boldsymbol{G}}^{-1} \\
&\partial_{\boldsymbol{\beta}^\top}[\boldsymbol{\Gamma}^\top\{\dot{\phi}^{-1}\boldsymbol{Z}^\top(\boldsymbol{y} - \tilde{\boldsymbol{\mu}}) - (\boldsymbol{I}_m \otimes \dot{\boldsymbol{G}}^{-1})\boldsymbol{b}(\boldsymbol{\beta})\}] \\
&\qquad = \boldsymbol{\Gamma}^\top\boldsymbol{Z}^\top\tilde{\boldsymbol{W}}\boldsymbol{X} + \boldsymbol{\Lambda} \odot \{\dot{\phi}^{-1}\boldsymbol{Z}^\top(\boldsymbol{y} - \tilde{\boldsymbol{\mu}}) - (\boldsymbol{I}_m \otimes \dot{\boldsymbol{G}}^{-1})\boldsymbol{b}(\boldsymbol{\beta})\} \\
&\partial_{\boldsymbol{b}(\boldsymbol{\beta})^\top}[\boldsymbol{\Gamma}^\top\{\dot{\phi}^{-1}\boldsymbol{Z}^\top(\boldsymbol{y} - \tilde{\boldsymbol{\mu}}) - (\boldsymbol{I}_m \otimes \dot{\boldsymbol{G}}^{-1})\boldsymbol{b}(\boldsymbol{\beta})\}] \\
&\qquad = \boldsymbol{\Gamma}^\top\boldsymbol{Z}^\top\tilde{\boldsymbol{W}}\boldsymbol{Z} - \boldsymbol{\Gamma}^\top(\boldsymbol{I}_m \otimes \dot{\boldsymbol{G}}^{-1}) + \boldsymbol{\Omega} \odot \{\dot{\phi}^{-1}\boldsymbol{Z}^\top(\boldsymbol{y} - \tilde{\boldsymbol{\mu}}) - (\boldsymbol{I}_m \otimes \dot{\boldsymbol{G}}^{-1})\boldsymbol{b}(\boldsymbol{\beta})\},
\end{aligned}$$

where $\boldsymbol{\Lambda}$ and $\boldsymbol{\Omega}$ are $p \times mp \times p$ and $p \times mp \times mp$ tensors respectively, given by

$$\begin{aligned}\boldsymbol{\Lambda}_{[,,k]} = [&-(\boldsymbol{X}_1^\top \tilde{\boldsymbol{W}}_1 \boldsymbol{X}_1 \dot{\boldsymbol{G}} + \boldsymbol{I}_p)^{-1} \boldsymbol{X}_1^\top \text{diag}(\boldsymbol{x}_{1\cdot k}) \tilde{\boldsymbol{W}}_1' \boldsymbol{X}_1 \dot{\boldsymbol{G}} (\boldsymbol{X}_1^\top \tilde{\boldsymbol{W}}_1 \boldsymbol{X}_1 \dot{\boldsymbol{G}} + \boldsymbol{I}_p)^{-1}, \ldots, \\ &- (\boldsymbol{X}_m^\top \tilde{\boldsymbol{W}}_m \boldsymbol{X}_m \dot{\boldsymbol{G}} + \boldsymbol{I}_p)^{-1} \boldsymbol{X}_m^\top \text{diag}(\boldsymbol{x}_{m\cdot k}) \tilde{\boldsymbol{W}}_m' \boldsymbol{X}_m \dot{\boldsymbol{G}} (\boldsymbol{X}_m^\top \tilde{\boldsymbol{W}}_m \boldsymbol{X}_m \dot{\boldsymbol{G}} + \boldsymbol{I}_p)^{-1}],\end{aligned}$$

for $k = 1, \ldots, p$, and

$$\boldsymbol{\Omega}_{[,(i-1)p+1:ip,(i-1)p+k]} = -(\boldsymbol{X}_i^\top \tilde{\boldsymbol{W}}_i \boldsymbol{X}_i \dot{\boldsymbol{G}} + \boldsymbol{I}_p)^{-1} \boldsymbol{X}_i^\top \text{diag}(\boldsymbol{x}_{i\cdot k}) \tilde{\boldsymbol{W}}_i' \boldsymbol{X}_i \dot{\boldsymbol{G}} (\boldsymbol{X}_i^\top \tilde{\boldsymbol{W}}_i \boldsymbol{X}_i \dot{\boldsymbol{G}} + \boldsymbol{I}_p)^{-1},$$

for $i = 1, \ldots, m$, $k = 1, \ldots, p$ and zero elsewhere. Here, $\boldsymbol{x}_{i\cdot k}$ is the n_i-vector formed by stacking the kth covariate values in the ith cluster. Notice these tensors are both $O_p(n^{-1})$ component-wise, and $\boldsymbol{\Omega}$ is sparse. The tensor product $\odot$ is defined as

$$\boldsymbol{\Omega} \odot \{\dot{\phi}^{-1} \boldsymbol{Z}^\top (\boldsymbol{y} - \tilde{\boldsymbol{\mu}}) - (\boldsymbol{I}_m \otimes \dot{\boldsymbol{G}}^{-1}) \boldsymbol{b}(\boldsymbol{\beta})\} = \sum_{l=1}^{mp} \boldsymbol{\Omega}_{[,l,]} \{\dot{\phi}^{-1} \boldsymbol{Z}^\top (\boldsymbol{y} - \tilde{\boldsymbol{\mu}}) - (\boldsymbol{I}_m \otimes \dot{\boldsymbol{G}}^{-1}) \boldsymbol{b}(\boldsymbol{\beta})\}_{[l]},$$

and

$$\begin{aligned}\boldsymbol{\Lambda} \odot \{\dot{\phi}^{-1} \boldsymbol{Z}^\top (\boldsymbol{y} - \tilde{\boldsymbol{\mu}}) - (\boldsymbol{I}_m \otimes \dot{\boldsymbol{G}}^{-1}) \boldsymbol{b}(\boldsymbol{\beta})\} &= \sum_{l=1}^{mp} \boldsymbol{\Lambda}_{[,l,]} \{\dot{\phi}^{-1} \boldsymbol{Z}^\top (\boldsymbol{y} - \tilde{\boldsymbol{\mu}}) - (\boldsymbol{I}_m \otimes \dot{\boldsymbol{G}}^{-1}) \boldsymbol{b}(\boldsymbol{\beta})\}_{[l]} \\ &= [\boldsymbol{\Omega} \odot \{\dot{\phi}^{-1} \boldsymbol{Z}^\top (\boldsymbol{y} - \tilde{\boldsymbol{\mu}}) - (\boldsymbol{I}_m \otimes \dot{\boldsymbol{G}}^{-1}) \boldsymbol{b}(\boldsymbol{\beta})\}] \boldsymbol{1}_{mp}.\end{aligned}$$

These are $p \times mp$ and $p \times p$ matrices respectively. Note also that

$$\boldsymbol{\Gamma}^\top \boldsymbol{Z}^\top \tilde{\boldsymbol{W}} \boldsymbol{X} = (\boldsymbol{\Gamma}^\top \boldsymbol{Z}^\top \tilde{\boldsymbol{W}} \boldsymbol{Z}) \boldsymbol{1}_{mp},$$

because $\boldsymbol{X}_i = \boldsymbol{Z}_i$. Let $\hat{\boldsymbol{\mu}} = \boldsymbol{\mu}(\hat{\boldsymbol{\theta}})$, and $\hat{\boldsymbol{\Gamma}}$, $\hat{\boldsymbol{W}}$ and $\hat{\boldsymbol{\Omega}}$ be $\boldsymbol{\Gamma}$, $\tilde{\boldsymbol{W}}$ and $\boldsymbol{\Omega}$ respectively with $\tilde{\boldsymbol{\theta}}$ replaced with $\hat{\boldsymbol{\theta}}$. From the above expressions and by conditions (C1)-(C2), we thus have

$$\begin{aligned}\nabla_{\boldsymbol{\beta}\boldsymbol{\beta}^\top} \ell_{PQL}(\hat{\boldsymbol{\beta}}) &= m\dot{\boldsymbol{G}}^{-1} + (\boldsymbol{1}_{mp}^\top \otimes \dot{\boldsymbol{G}}^{-1}) \hat{\boldsymbol{\Gamma}} + \hat{\boldsymbol{\Gamma}}^\top (\boldsymbol{I}_m \otimes \dot{\boldsymbol{G}}^{-1}) \boldsymbol{1}_{mp} - \hat{\boldsymbol{\Gamma}}^\top (\boldsymbol{I}_m \otimes \dot{\boldsymbol{G}}^{-1}) \hat{\boldsymbol{\Gamma}} + \hat{\boldsymbol{\Gamma}}^\top \boldsymbol{Z}^\top \hat{\boldsymbol{W}} \boldsymbol{Z} \hat{\boldsymbol{\Gamma}} \\ &\quad + [\hat{\boldsymbol{\Omega}} \odot \{\dot{\phi}^{-1} \boldsymbol{Z}^\top (\boldsymbol{y} - \hat{\boldsymbol{\mu}}) - (\boldsymbol{I}_m \otimes \dot{\boldsymbol{G}}^{-1}) \boldsymbol{b}(\hat{\boldsymbol{\beta}})\}] \hat{\boldsymbol{\Gamma}} \\ &= m\dot{\boldsymbol{G}}^{-1} + O_p(n_L^{-1} m) + O_p(n_L^{-1} m) + O_p(n_L^{-2} m) + O_p(n_L^{-1} m) + O_p(n_L^{-1} m) \\ &= m\dot{\boldsymbol{G}}^{-1} + O_p(n_L^{-1} m),\end{aligned}$$

since $\hat{\boldsymbol{\Omega}} \odot \{\dot{\phi}^{-1} \boldsymbol{Z}^\top (\boldsymbol{y} - \hat{\boldsymbol{\mu}}) - (\boldsymbol{I}_m \otimes \dot{\boldsymbol{G}}^{-1}) \boldsymbol{b}(\hat{\boldsymbol{\beta}})\} = O_p(1)$, $\|\hat{\boldsymbol{\Omega}} \odot \{\dot{\phi}^{-1} \boldsymbol{Z}^\top (\boldsymbol{y} - \hat{\boldsymbol{\mu}}) - (\boldsymbol{I}_m \otimes \dot{\boldsymbol{G}}^{-1}) \boldsymbol{b}(\hat{\boldsymbol{\beta}})\}\|_F = O_p(m^{1/2})$, and $\|\hat{\boldsymbol{\Gamma}}\|_F = O_p(n_L^{-1} m^{1/2})$ so that $\|[\hat{\boldsymbol{\Omega}} \odot \{\dot{\phi}^{-1} \boldsymbol{Z}^\top (\boldsymbol{y} - \hat{\boldsymbol{\mu}}) - (\boldsymbol{I}_m \otimes$

$\dot{\boldsymbol{G}}^{-1})\boldsymbol{b}(\hat{\boldsymbol{\beta}})\}]\hat{\boldsymbol{\Gamma}}\|_F = O_p(n_L^{-1}m)$ by the Cauchy-Schwarz inequality, where $\|\cdot\|_F$ denotes the Frobenius norm. Therefore

$$m^{-1}\nabla_{\boldsymbol{\beta}\boldsymbol{\beta}^\top}\ell_{PQL}(\hat{\boldsymbol{\beta}}) = \dot{\boldsymbol{G}}^{-1} + O_p(n_L^{-1})$$

and the result then follows from the continuous mapping theorem.

□

Lemma 1 is not surprising given the results of Ning et al. (2024), which prove that the asymptotic variance of $\sqrt{m}(\hat{\boldsymbol{\beta}} - \dot{\boldsymbol{\beta}})$ is $\dot{\boldsymbol{G}}$. Since the Laplace approximation approximates the (marginal) likelihood with a relative error of order $O(n_L^{-1})$, then it is reasonable from the independence of clusters and classic maximum likelihood estimation results (McCulloch & Searle, 2004) - which apply for any fixed n_L as long as m grows - that $m\{-\partial_{\boldsymbol{\beta}\boldsymbol{\beta}^\top}\ell_{PQL}(\hat{\boldsymbol{\beta}})\}^{-1}$ tends to the asymptotic variance of $\hat{\boldsymbol{\beta}}$, which is $\dot{\boldsymbol{G}}$.

A.1 Proof of Theorem 1

From Lemma 1, we know that $m\{-\partial_{\boldsymbol{\beta}\boldsymbol{\beta}^\top}\ell_{PQL}(\hat{\boldsymbol{\beta}})\}^{-1} = \dot{\boldsymbol{G}} + O_p(n_L^{-1})$. As such, we can simplify equation (6) in the main text as

$$\hat{V}(\hat{\boldsymbol{\theta}} - \dot{\boldsymbol{\theta}}) = \begin{bmatrix} 0 & 0 \\ 0 & (\boldsymbol{Z}^\top\hat{\boldsymbol{W}}\boldsymbol{Z})^{-1} \end{bmatrix} + \begin{bmatrix} \boldsymbol{I} \\ -\boldsymbol{1}_m \otimes \boldsymbol{I}_p \end{bmatrix} m^{-1}\dot{\boldsymbol{G}} \begin{bmatrix} \boldsymbol{I} & -\boldsymbol{1}_m^\top \otimes \boldsymbol{I}_p \end{bmatrix} + O_p(n_L^{-1}m^{-1}). \tag{3}$$

For the ith prediction gap $\hat{\boldsymbol{b}}_i - \dot{\boldsymbol{b}}_i$, equation (5) in the main text gives

$$\hat{V}_{\boldsymbol{b},\boldsymbol{y}}(\boldsymbol{b}(\hat{\boldsymbol{\psi}}) - \dot{\boldsymbol{b}}_i) = m^{-1}\dot{\boldsymbol{G}} + (\boldsymbol{Z}_i^\top\hat{\boldsymbol{W}}_i\boldsymbol{Z}_i)^{-1} + O_p(n_L^{-1}m^{-1}),$$

while from equation (1) we obtain

$$V_{(\dot{\boldsymbol{b}},\boldsymbol{y})|\dot{\boldsymbol{b}}_i}(\hat{\boldsymbol{b}}_i - \dot{\boldsymbol{b}}_i) = m^{-1}\dot{\boldsymbol{G}} + (\boldsymbol{Z}_i^\top\dot{\boldsymbol{W}}_i\boldsymbol{Z}_i)^{-1} + O_p(n_L^{-1}m^{-1}),$$

and

$$V_{\dot{\boldsymbol{b}},\boldsymbol{y}}(\hat{\boldsymbol{b}}_i - \dot{\boldsymbol{b}}_i) = m^{-1}\dot{\boldsymbol{G}} + E_{\dot{\boldsymbol{b}},\boldsymbol{y}}\{(\boldsymbol{Z}_i^\top\dot{\boldsymbol{W}}_i\boldsymbol{Z}_i)^{-1}\} + O_p(n_L^{-1}m^{-1}).$$

Next, from Ning et al. (2024) we have that, conditional on $\dot{\boldsymbol{b}}_i$, $\hat{\boldsymbol{b}}_i \xrightarrow{p} \dot{\boldsymbol{b}}_i$. Thus by the continuous mapping theorem $n_i(\boldsymbol{Z}_i^\top \hat{\boldsymbol{W}}_i \boldsymbol{Z}_i)^{-1} \xrightarrow{p} \lim_{n_i \to \infty} n_i(\boldsymbol{Z}_i^\top \dot{\boldsymbol{W}}_i \boldsymbol{Z}_i)^{-1}$. Therefore, we have

$$\begin{aligned}\hat{V}_{\dot{\boldsymbol{b}},\boldsymbol{y}}(\boldsymbol{b}(\hat{\psi}) - \dot{\boldsymbol{b}}_i) - V_{(\dot{\boldsymbol{b}},\boldsymbol{y})|\dot{\boldsymbol{b}}_i}(\hat{\boldsymbol{b}}_i - \dot{\boldsymbol{b}}_i) &= (\boldsymbol{Z}_i^\top \hat{\boldsymbol{W}}_i \boldsymbol{Z}_i)^{-1} - (\boldsymbol{Z}_i^\top \dot{\boldsymbol{W}}_i \boldsymbol{Z}_i)^{-1} + O_p(n_L^{-1} m^{-1}) \\ &= o_p(n^{-1}) + O_p(n_L^{-1} m^{-1}) \\ &= o_p\{V_{(\dot{\boldsymbol{b}},\boldsymbol{y})|\dot{\boldsymbol{b}}_i}(\hat{\boldsymbol{b}}_i - \dot{\boldsymbol{b}}_i)\}.\end{aligned}$$

This last result implies that the `glmmTMB` estimator for a particular cluster is a consistent estimator of the conditional variance of the prediction gap, conditional on the random effects of that cluster $\dot{\boldsymbol{b}}_i$.

A.2 Proof of Theorem 2

We examine the behaviour of normalised versions of the two main terms in equation (1) in the main text, conditional on $\dot{\boldsymbol{b}}_i$. First, consider the case $m n_L^{-1} \to \gamma \in (0, \infty)$. We write

$$-m^{-1/2} \sum_{j=1}^{m} \dot{\boldsymbol{b}}_j = -m^{-1/2} \sum_{j=1, j \neq i}^{m} \dot{\boldsymbol{b}}_j - m^{-1/2} \dot{\boldsymbol{b}}_i = -m^{-1/2} \sum_{j=1, j \neq i}^{m} \dot{\boldsymbol{b}}_j + o(m^{-1/2}).$$

Since $\dot{\boldsymbol{b}}_j$ is independent and identically distributed between clusters, then $-m^{-1/2} \sum_{j=1, j\neq i}^{m} \dot{\boldsymbol{b}}_j \xrightarrow{d} N(\boldsymbol{0}, \dot{\boldsymbol{G}})$ and therefore so does $-m^{-1/2} \sum_{j=1}^{m} \dot{\boldsymbol{b}}_j$. Next, Ning et al. (2024) prove under conditions (C1)-(C4) that

$$n_i^{1/2} (\boldsymbol{Z}_i^\top \dot{\boldsymbol{W}}_i \boldsymbol{Z}_i)^{-1} [\boldsymbol{Z}_i^\top \{\boldsymbol{y}_i - \boldsymbol{\mu}_i(\dot{\boldsymbol{\theta}})\}] \xrightarrow{d} N\{\boldsymbol{0}, \dot{\boldsymbol{K}}_i\}.$$

From the conditional independence of $-m^{-1} \sum_{j=1, j\neq i}^{m} \dot{\boldsymbol{b}}_j$ and $(\boldsymbol{Z}_i^\top \dot{\boldsymbol{W}}_i \boldsymbol{Z}_i)^{-1} [\boldsymbol{Z}_i^\top \{\boldsymbol{y}_i - \boldsymbol{\mu}_i(\dot{\boldsymbol{\theta}})\}]$ given $\dot{\boldsymbol{b}}_i$, we thus know from equation (1) that

$$\hat{\boldsymbol{b}}_i - \dot{\boldsymbol{b}}_i \xrightarrow{D} N(\boldsymbol{0}, m^{-1} \dot{\boldsymbol{G}}) * N\{\boldsymbol{0}, (\boldsymbol{Z}_i^\top \dot{\boldsymbol{W}}_i \boldsymbol{Z}_i)^{-1}\},$$

where '$*$' denotes the convolution operator. Part b of the theorem follows from this. On the other hand, when $m n_L^{-1} \to 0$, $-m^{-1} \sum_{i=1}^{m} \dot{\boldsymbol{b}}_i$ is the leading term on the right hand side of (1), and when $m n_U^{-1} \to \infty$, $(\boldsymbol{Z}_i^\top \dot{\boldsymbol{W}}_i \boldsymbol{Z}_i)^{-1} [\boldsymbol{Z}_i^\top \{\boldsymbol{y}_i - \boldsymbol{\mu}_i(\dot{\boldsymbol{\theta}})\}]$ is the leading term. Parts a and c of the theorem follow from this.

A.3 Proof of Corollary 1

We know from Theorem 2 that, conditional on the random effects $\dot{\boldsymbol{b}}_i$ and for each $i = 1, \ldots, m$,

$$\lim_{m,n_L\to\infty} P[\boldsymbol{z}^{*\top}\hat{\boldsymbol{b}}_i + \Phi^{-1}(\alpha/2)\{\boldsymbol{z}^{*\top}V_{(\dot{\boldsymbol{b}},\boldsymbol{y})|\dot{\boldsymbol{b}}_i}(\hat{\boldsymbol{b}}_i - \dot{\boldsymbol{b}}_i)\boldsymbol{z}^*\}^{1/2} < \boldsymbol{z}^{*\top}\dot{\boldsymbol{b}}_i < \boldsymbol{z}^{*\top}\hat{\boldsymbol{b}}_i + \Phi^{-1}(1-\alpha/2)\{\boldsymbol{z}^{*\top}V_{(\dot{\boldsymbol{b}},\boldsymbol{y})|\dot{\boldsymbol{b}}_i}(\hat{\boldsymbol{b}}_i - \dot{\boldsymbol{b}}_i)\boldsymbol{z}^*\}^{1/2}|\dot{\boldsymbol{b}}_i] = 1-\alpha$$

from the definition of convergence in distribution. Thus from Theorem 1 and Slutsky's Theorem, we have

$$\lim_{m,n_L\to\infty} P[\boldsymbol{z}^{*\top}\hat{\boldsymbol{b}}_i + \Phi^{-1}(\alpha/2)\{\boldsymbol{z}^{*\top}\hat{V}(\hat{\boldsymbol{b}}_i - \dot{\boldsymbol{b}}_i)\boldsymbol{z}^*\}^{1/2} < \boldsymbol{z}^{*\top}\dot{\boldsymbol{b}}_i < \boldsymbol{z}^{*\top}\hat{\boldsymbol{b}}_i + \Phi^{-1}(1-\alpha/2)\{\boldsymbol{z}^{*\top}\hat{V}(\hat{\boldsymbol{b}}_i - \dot{\boldsymbol{b}}_i)\boldsymbol{z}^*\}^{1/2}|\dot{\boldsymbol{b}}_i] = 1-\alpha.$$

Finally, for the unconditional result, we note that $P[\boldsymbol{z}^{*\top}\hat{\boldsymbol{b}}_i + \Phi^{-1}(\alpha/2)\{\boldsymbol{z}^{*\top}\hat{V}(\hat{\boldsymbol{b}}_i - \dot{\boldsymbol{b}}_i)\boldsymbol{z}^*\}^{1/2} < \boldsymbol{z}^{*\top}\dot{\boldsymbol{b}}_i < \boldsymbol{z}^{*\top}\hat{\boldsymbol{b}}_i + \Phi^{-1}(1-\alpha/2)\{\boldsymbol{z}^{*\top}\hat{V}(\hat{\boldsymbol{b}}_i - \dot{\boldsymbol{b}}_i)\boldsymbol{z}^*\}^{1/2}|\dot{\boldsymbol{b}}_i]f(\dot{\boldsymbol{b}}_i)$ is bounded by $f(\dot{\boldsymbol{b}}_i)$. Hence applying $\int f(\dot{\boldsymbol{b}}_i)d\dot{\boldsymbol{b}}_i = 1$ and the dominated convergence theorem, we obtain

$$\begin{aligned}
&\lim_{m,n_L\to\infty} P[\boldsymbol{z}^{*\top}\hat{\boldsymbol{b}}_i + \Phi^{-1}(\alpha/2)\{\boldsymbol{z}^{*\top}\hat{V}(\hat{\boldsymbol{b}}_i - \dot{\boldsymbol{b}}_i)\boldsymbol{z}^*\}^{1/2} < \boldsymbol{z}^{*\top}\dot{\boldsymbol{b}}_i < \\
&\boldsymbol{z}^{*\top}\hat{\boldsymbol{b}}_i + \Phi^{-1}(1-\alpha/2)\{\boldsymbol{z}^{*\top}\hat{V}(\hat{\boldsymbol{b}}_i - \dot{\boldsymbol{b}}_i)\boldsymbol{z}^*\}^{1/2}] \\
=&\lim_{m,n_L\to\infty}\int P[\boldsymbol{z}^{*\top}\hat{\boldsymbol{b}}_i + \Phi^{-1}(\alpha/2)\{\boldsymbol{z}^{*\top}\hat{V}(\hat{\boldsymbol{b}}_i - \dot{\boldsymbol{b}}_i)\boldsymbol{z}^*\}^{1/2} < \boldsymbol{z}^{*\top}\dot{\boldsymbol{b}}_i < \\
&\boldsymbol{z}^{*\top}\hat{\boldsymbol{b}}_i + \Phi^{-1}(1-\alpha/2)\{\boldsymbol{z}^{*\top}\hat{V}(\hat{\boldsymbol{b}}_i - \dot{\boldsymbol{b}}_i)\boldsymbol{z}^*\}^{1/2}|\dot{\boldsymbol{b}}_i]f(\dot{\boldsymbol{b}}_i)d\dot{\boldsymbol{b}}_i \\
=&\int\lim_{m,n_L\to\infty} P[\boldsymbol{z}^{*\top}\hat{\boldsymbol{b}}_i + \Phi^{-1}(\alpha/2)\{\boldsymbol{z}^{*\top}\hat{V}(\hat{\boldsymbol{b}}_i - \dot{\boldsymbol{b}}_i)\boldsymbol{z}^*\}^{1/2} < \boldsymbol{z}^{*\top}\dot{\boldsymbol{b}}_i < \\
&\boldsymbol{z}^{*\top}\hat{\boldsymbol{b}}_i + \Phi^{-1}(1-\alpha/2)\{\boldsymbol{z}^{*\top}\hat{V}(\hat{\boldsymbol{b}}_i - \dot{\boldsymbol{b}}_i)\boldsymbol{z}^*\}^{1/2}|\dot{\boldsymbol{b}}_i]f(\dot{\boldsymbol{b}}_i)d\dot{\boldsymbol{b}}_i \\
=&\int(1-\alpha)f(\dot{\boldsymbol{b}}_i)d\dot{\boldsymbol{b}}_i \\
=&1-\alpha.
\end{aligned}$$

B Supplementary bolus example results

Table 1: Parameter estimates produced by `glmmTMB` and `lme4` for the bolus example

	`lme4`	`glmmTMB`
β_0	2.074	2.074
β_1	-0.067	-0.067
$\sqrt{\text{Var}(b_{i0})}$	0.591	0.591
$\sqrt{\text{Var}(b_{i1})}$	0.079	0.079
$\text{corr}(b_{i0}, b_{i1})$	-0.52	-0.52

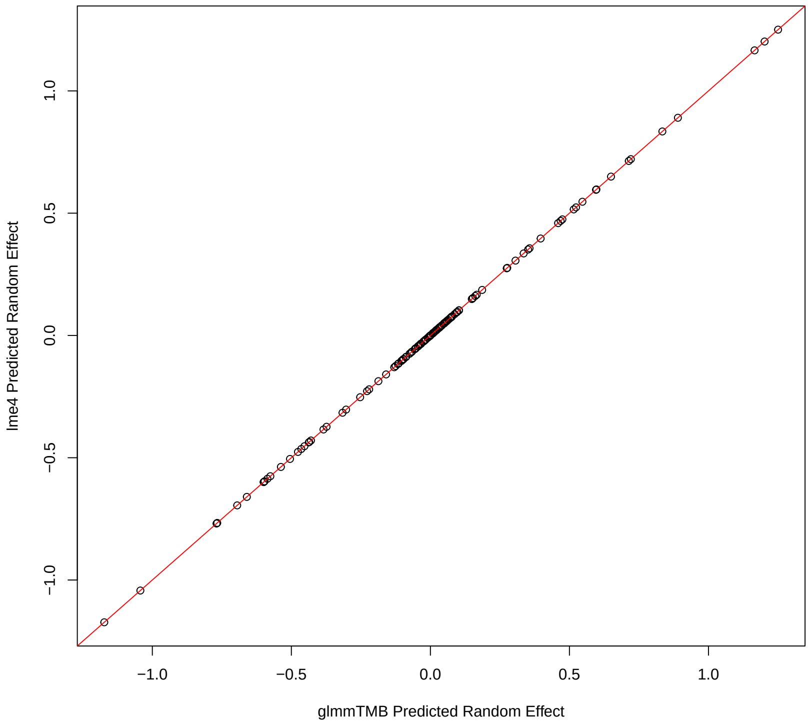

Figure 1: *Comparison of `glmmTMB` and `lme4` point predictions of the random effects.*

C Supplementary simulation results

From Table 2, the estimated variance of the fixed effects tends towards $m^{-1}\dot{\boldsymbol{G}}$, consistent with the theory in Ning et al. (2024). Table 3 shows that the mean (over 1000 simulations) absolute difference between the Laplace estimates produced by `glmmTMB` and the PQL estimates for $\dot{\boldsymbol{b}}_1$

become very small as (m, n) grow, and in particular is negligible when $n = 100$ or more, even for small values of m.

Table 2: Estimated variance for the fixed effects, using the `glmmTMB` package.

		Intercept				Slope			
	m	$n=25$	$n=50$	$n=100$	$n=200$	$n=25$	$n=50$	$n=100$	$n=200$
glmmTMB	25	1.036	1.018	1.010	1.005	1.028	1.014	1.007	1.003
	50	1.035	1.019	1.010	1.005	1.029	1.015	1.007	1.003
	100	1.035	1.018	1.009	1.005	1.030	1.014	1.007	1.003
	200	1.035	1.018	1.009	1.005	1.029	1.014	1.007	1.003

Table 3: Mean absolute differences between the PQL and TMB estimates of the random effects $\dot{\boldsymbol{b}}_1$.

	Random Intercept				Random Slope			
m	$n=25$	$n=50$	$n=100$	$n=200$	$n=25$	$n=50$	$n=100$	$n=200$
25	0.0353	0.0040	0.0000	0.0000	0.0051	0.0006	0.0000	0.0000
50	0.0363	0.0014	0.0000	0.0000	0.0042	0.0002	0.0000	0.0000
100	0.0381	0.0001	0.0000	0.0000	0.0038	0.0000	0.0000	0.0000
200	0.0379	0.0000	0.0000	0.0000	0.0032	0.0000	0.0000	0.0000

D Supplementary results for the Poisson intercept-only example

We compare the length of the correct unconditional intervals against the length of naive intervals using the true (asymptotic) unconditional variance $\exp(-\dot{\beta})\exp(0.5\dot{\sigma}_b^2)$ plus a normal assumption. This latter interval is the interval that would be constructed by `glmmTMB` if it correctly estimated the true unconditional variance of the prediction gap, and does not in general have correct nominal coverage.

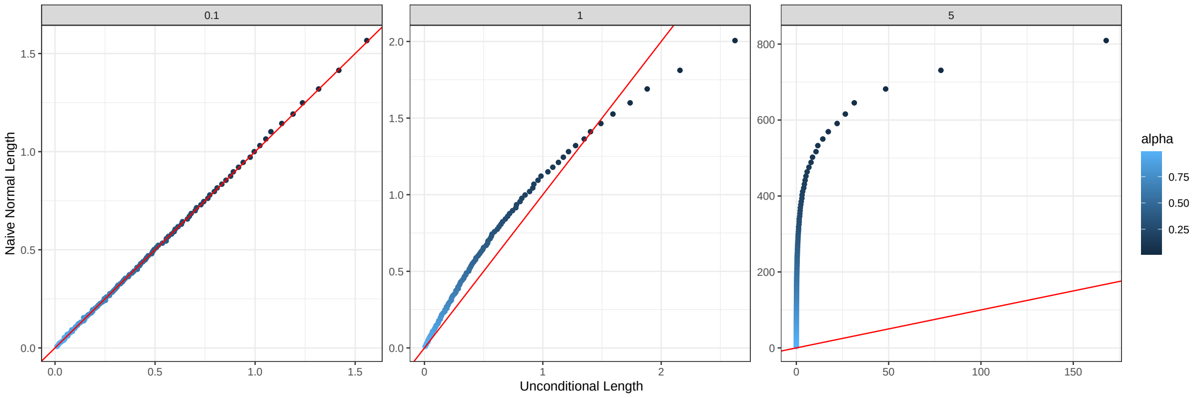

Figure 2: Naive interval lengths versus unconditional interval lengths, for $\dot{\sigma}_b \in \{0.1, 1, 5\}$.

We see that the shape of the graphs in the lower panel are similar to that of Figure 2, but the naive interval lengths become overly large as $\dot{\sigma}_b^2$ grows, resulting in overly conservative intervals.

E Approximating U_2 with U_3

To motivate approximating U_2 with U_3, we draw a parallel to the UMSEP derivation of Kackar and Harville (1984). If we left and right-multiply U_2 by an arbitrary mp_r-vector of constants $\boldsymbol{c}$, then we have

$$\boldsymbol{c}^\top U_2 \boldsymbol{c} = E_{\boldsymbol{y}}(\boldsymbol{c}^\top [\partial_{\boldsymbol{b}\boldsymbol{b}^\top} l\{\boldsymbol{b}(\dot{\boldsymbol{\psi}}), \dot{\boldsymbol{\psi}}\}]^{-1} \partial_{\boldsymbol{b}\boldsymbol{\psi}^\top} l\{\boldsymbol{b}(\dot{\boldsymbol{\psi}}), \dot{\boldsymbol{\psi}}\}(\hat{\boldsymbol{\psi}} - \dot{\boldsymbol{\psi}})$$
$$(\hat{\boldsymbol{\psi}} - \dot{\boldsymbol{\psi}})^\top [\partial_{\boldsymbol{b}\boldsymbol{\psi}^\top} l\{\boldsymbol{b}(\dot{\boldsymbol{\psi}}), \dot{\boldsymbol{\psi}}\}]^\top [\partial_{\boldsymbol{b}\boldsymbol{b}^\top} l\{\boldsymbol{b}(\dot{\boldsymbol{\psi}}), \dot{\boldsymbol{\psi}}\}]^{-1} \boldsymbol{c} + O_p(m^{-1})).$$

Assume the smaller order term has finite expectation and thus negligible. Furthermore, treat $\boldsymbol{b}(\boldsymbol{\psi})$ as if it was not a function of $\boldsymbol{y}$, and assume $E_{\dot{\boldsymbol{b}},\boldsymbol{y}}(\hat{\boldsymbol{\psi}} - \dot{\boldsymbol{\psi}}) = \boldsymbol{0}$. Similar to Kackar and Harville (1984) then, we can approximate $\boldsymbol{c}^\top U_2 \boldsymbol{c}$ as

$$\mathrm{tr}([\partial_{\boldsymbol{b}\boldsymbol{\psi}^\top} l\{\boldsymbol{b}(\dot{\boldsymbol{\psi}}), \dot{\boldsymbol{\psi}}\}]^\top [\partial_{\boldsymbol{b}\boldsymbol{b}^\top} l\{\boldsymbol{b}(\dot{\boldsymbol{\psi}}), \dot{\boldsymbol{\psi}}\}]^{-1} \boldsymbol{c}\boldsymbol{c}^\top [\partial_{\boldsymbol{b}\boldsymbol{b}^\top} l\{\boldsymbol{b}(\dot{\boldsymbol{\psi}}), \dot{\boldsymbol{\psi}}\}]^{-1} \partial_{\boldsymbol{b}\boldsymbol{\psi}^\top} l\{\boldsymbol{b}(\dot{\boldsymbol{\psi}}), \dot{\boldsymbol{\psi}}\} \mathrm{cov}(\hat{\boldsymbol{\psi}} - \dot{\boldsymbol{\psi}})).$$

Finally, if we replace $[\partial_{\boldsymbol{b}\boldsymbol{\psi}^\top} l\{\boldsymbol{b}(\dot{\boldsymbol{\psi}}), \dot{\boldsymbol{\psi}}\}]^\top [\partial_{\boldsymbol{b}\boldsymbol{b}^\top} l\{\boldsymbol{b}(\dot{\boldsymbol{\psi}}), \dot{\boldsymbol{\psi}}\}]^{-1} \boldsymbol{c}\boldsymbol{c}^\top [\partial_{\boldsymbol{b}\boldsymbol{b}^\top} l\{\boldsymbol{b}(\dot{\boldsymbol{\psi}}), \dot{\boldsymbol{\psi}}\}]^{-1} \partial_{\boldsymbol{b}\boldsymbol{\psi}^\top} l\{\boldsymbol{b}(\dot{\boldsymbol{\psi}}), \dot{\boldsymbol{\psi}}\}$ by $\mathrm{cov}([\partial_{\boldsymbol{b}\boldsymbol{\psi}^\top} l\{\boldsymbol{b}(\dot{\boldsymbol{\psi}}), \dot{\boldsymbol{\psi}}\}]^\top [\partial_{\boldsymbol{b}\boldsymbol{b}^\top} l\{\boldsymbol{b}(\dot{\boldsymbol{\psi}}), \dot{\boldsymbol{\psi}}\}]^{-1} \boldsymbol{c})$, then we essentially arrive at the UMSEP estimator derived by Kackar and Harville (1984). While there is a closed-form expression for this covariance in LMMs based on Henderson's mixed model equations (Kackar & Harville, 1984), such a closed-form expression does not exist the case of GLMMs. Although it may be possible to circumvent this e.g., by using an LMM approximation to the GLMM as in González-Manteiga et al. (2007) and Saei and Chambers (2003), the consequences of this are unclear. Moreover, while the trace identity can be applied to the scalar quantity $\boldsymbol{c}^\top U_2 \boldsymbol{c}$, it cannot be used when we are interested in a vector of prediction gaps, i.e., it cannot be applied to U_2 directly when $p_r > 1$.
