# Supplementary material for: Inferential procedures for random effects in generalized linear mixed models
Source: PLoS One. 2025 Apr 16;20(4):e0320797. doi: 10.1371/journal.pone.0320797 (PMC12002512; doi:10.1371/journal.pone.0320797)
Supplement: Attachment — Submitted filename: response_to_reviewers.pdf [file pone.0320797.s004.pdf]

Manuscript PONE-D-24-60152

We appreciate the careful feedback provided by the two reviewers, which have been useful in strengthening the clarity and methodological contributions of the manuscript. In response, we have made a number of changes as part of our revised submission, which are highlighted in blue. Our point-by-point response to each of the reviewers' comments is provided below.

Response to Reviewer One

Comment 1: *Page 2, Line 69: please, use j as subscript,* β_{1j} *and* b_{i1j}.

Response: Thank you for pointing this out. Note the subscript 'j' here is used to denote the time period covariate, i.e., $\ln(\mu_{ij}) = \beta_0 + \beta_1 x_{ij} + b_{i0} + b_{i1} x_{ij}$, where $x_{ij} = j$. In independent cluster GLMMs such as this one, the fixed and random slopes for time do not usually also vary with time, and so it is sensible for them to not involve a subscript 'j' (e.g., Hui et al., 2017; Neuhaus et al., 2013). We have made modifications on page 2 line 69 to make this point clearer.

Comment 2: *Page 2, Line 69:* β_{1j} *instead* β_1.

Response: Similar to comment 1, β_1 is the fixed slope for time, and in most applications of independent cluster GLMMs we typically do not let this vary with j. We have made changes at page 2 line 69 to clarify this point.

Comment 3: *Page 4, Line 145: The meaning of n here is unclear. Does it refer to the mean sample size? It would be helpful to define this value explicitly.*

Response: Thank you for the suggestion. Here, n indeed refers to the mean cluster size; we have now made clear in page 4 line 145 of the revised manuscript.

Comment 4: *Page 4, Line 157: I am unsure whether* Ξ *is well defined. Is it a matrix obtained by joining the vectors in columns and then transposing, or is it a vector resulting from joining the vectors? If it's the latter, the transposes should be inside the brackets, not outside.*

Response: Thank you for pointing this out. The reviewer is correct that it is the former case i.e., the matrix obtained by joining the vectors in columns and then transposing. We have now clarified this by including the matrix dimensions in line 157 page 4 of the revised version of the manuscript.

Comment 5: *Page 4, Line 161: Could you clarify what "bdiag" refers to?*

Response: We have updated the revised manuscript in line 161 to include the definition of bdiag$(\cdot)$ as the block diagonal operator i.e., takes one or more matrices and forms an overall block diagonal matrix with the smaller matrices as the diagonal components.

Comment 6: *Page 5, Line 166: What is the "half vectorization operator"? A brief explanation would be helpful.*

Response: Thank you for the suggestion. The half vectorization operator stacks elements from the lower triangular portion (including the diagonal) of a (symmetric) matrix, column-wise, into a vector. We have now included this explanation in page 5 line 166 of the revised manuscript.

Comment 6: *Page 17, Lines 654 and 661: Please unify the notation for numbers, i.e., use either 1,000 or 1000, and 10,000 or 10000 consistently.*

Response: We have unified our notation in page 17 lines 654 and 661 in the revised manuscript to not use commas.

Response to Reviewer Two

Comment 1: *The manuscript is very technical. I would advocate the main text to contain the major results and the remainder (derivations and proofs) to be put in the appendix.*

Response: Thank for this useful suggestion. In the revised version of the manuscript, we have moved the technical conditions into the supplementary material as advised by the reviewer, while keeping the major results in the main text. However, we have kept some derivations that we believe are crucial to understanding the intuition behind our major results in the main text, such as the main steps in the derivations of CMSEP, UMSEP, the glmmTMB estimator, and the Poisson intercept-only GLMM example; we feel they add clarity to the exposition and form part of the main contributions of the manuscript.

Comment 2: *In practical applications, e.g. in longitudinal studies, the cluster sizes are usually much smaller than the sample sizes. Can the authors comment on both the "glmmTMB" and "lme4" estimation properties in the cases, where there is an order of magnitude difference between the cluster sizes and the sample sizes? For example, cluster size = 5, 10 and sample size = 50, 100?*

Response: This is an insightful point. First, we note that the conditions underlying our theoretical developments suggest an order of magnitude difference between m and n is unlikely to greatly deteriorate the asymptotic approximations which `lme4` and `glmmTMB` essentially use, so long as the *actual values* of the cluster sizes themselves (regardless of the ratio to the number of clusters) are sufficiently large for the asymptotic approximations to be accurate. This is based on our theory requiring $mn^{-2} \to 0$ i.e., the number of clusters are not growing faster than the cluster size squared, and indeed our numerical results support this, e.g., coverage of the `glmmTMB` intervals are close to nominal when $m = 200$ and $n = 25$. This is also consistent with recent simulation results in, e.g., Lyu and Welsh, 2021a, 2021b, who consider even smaller sample cluster sizes of $n = 10$. Our theory and simulation results further suggest the coverage of `lme4` random effects prediction intervals tend toward nominal (from undercoverage) even in the partnered case, as the ratio of m/n increases.

To further the above point, in the appendix of this response to reviewers document we have con-

ducted some additional simulations using the same setup as in the main text, but at the smallest $n = 5$ and $m = 50$ case suggested by the reviewer. The results of these are largely consistent with those already in the main text, except the unconditional intervals exhibit stronger overcoverage, and all intervals are noticeably but expectedly wider. This indicates our asymptotic theory can still be applicable at a relatively small but practical cluster and sample size of $n = 5$ and $m = 50$, respectively, at least for the Poisson case with the given parameter values. We have now included a summary of the above discussion in the final section of the revised manuscript.

As an aside, we do note however when the actual cluster sizes are extremely small, the Laplace approximation/penalized quasi-likelihood and thus point estimation itself could be quite inaccurate. This is true especially for cases such as binary and low count Poisson response GLMMs (e.g., Hui, 2021; Vonesh, 1996). Although this problem did not substantially affect our simulations, it nevertheless means that caution must be taken when applying asymptotic approximations to small datasets (despite the reasonable numerical results discussed above).

Finally, we wish to reiterate that although very small cluster sizes are important in much real data, there are increasingly many many settings in practice where cluster sizes are not that small relative to the number of clusters, and for which the asymptotic approximations and large sample results established in this manuscript are accurate and relevant, e.g., educational studies with large numbers of students (units) grouped within schools (clusters) (e.g., Hui et al., 2019), medical studies with large groups (clusters) of patients (units) treated at different hospitals (e.g., Pratesi, 2016), and settings where the data for each cluster are recorded at relatively high temporal frequency (Asparouhov & Muthén, 2020; Ployhart et al., 2025). We have reiterated this point in Section 3.1 of the revised manuscript.

Comment 3: *How will the finite sample results be affected by missingness patterns, e.g. MAR?*

Response: This is an interesting question. Although our theoretical framework is currently not set up to incorporate any missingness in the response or fixed/random effect covariates, the fact that we allow uneven cluster sizes suggests that, as a starting point, we could rely on standard

literature in the case of Missing Completely at Random (MCAR) and perform complete case analysis without any theoretical properties changing greatly (Ibrahim and Molenberghs, 2009; Laird, 1988). On the other hand, we conjecture there are likely to be biases in both point and interval estimates if our major theoretical results are directly applied to the case of (Missing Not at Random) MNAR responses and/or covariates; this would be consistent with the standard literature on MNAR in the context of independent cluster mixed models (e.g., Ibrahim et al., 2001; Wu & Wu, 2007).

Turning to the case of (Missing at Random) MAR data, a common approach is to employ some form of multiple imputation (Little and Rubin, 2019). We conjecture that our asymptotic developments conditional on $\boldsymbol{b}_i$ e.g., Theorems 1 and 2, will still be valid for fixed effects (noting the $\boldsymbol{b}_i$ are under this setting) inference if classic methods of imputation, such as using Rubin's rules and increasing the variance of the asymptotic approximating normal distribution, are employed (Huque et al., 2018). However, further theoretical and numerical investigation is required when it comes to random effects inference/prediction intervals in the unconditional case, since the extra variability from imputation would need to be incorporated into our theoretical results either within the normal mixture distribution component or the normal distribution component of the asymptotic approximating distribution; again this could result in non-standard distributions of the prediction gap.

We have now included a summary of the discussion in the final section of the revised manuscript, highlighting the extension of our theoretical and finite sample results to the case of various missingness patterns and assumptions as an important avenue of future research.

Comment 4: *Please check the manuscript for spelling and grammatical errors.*

Response: Thank you for the comment. We have done our best to go through the revised manuscript in detail checking for spelling and grammatical errors, and fixed some typos as a result.

Comment 5: *Supp. B, Table 1 Is there a possible error in reporting the variance estimates of*

the random slope effects?

Response: Thank you for catching this. We have corrected the error in Table 1 in the revised version of the supplementary material.

References

Asparouhov, T., & Muthén, B. (2020). Comparison of models for the analysis of intensive longitudinal data. *Structural Equation Modeling: A Multidisciplinary Journal*, *27*, 275–297.

Hui, F. K. C., Müller, S., & Welsh, A. H. (2019). Testing random effects in linear mixed models: another look at the F-test (with discussion). *Australian & New Zealand Journal of Statistics*, *61*, 61–84.

Hui, F. K. C. (2021). On the use of a penalized quasilikelihood information criterion for generalized linear mixed models. *Biometrika*, *108*, 353–365.

Hui, F. K. C., Müller, S., & Welsh, A. H. (2017). Hierarchical selection of fixed and random effects in generalized linear mixed models. *Statistica Sinica*, *27*, 501–518.

Huque, M. H., Carlin, J. B., Simpson, J. A., & Lee, K. J. (2018). A comparison of multiple imputation methods for missing data in longitudinal studies. *BMC medical research methodology*, *18*, 1–16.

Ibrahim, J. G., Chen, M.-H., & Lipsitz, S. R. (2001). Missing responses in generalised linear mixed models when the missing data mechanism is nonignorable. *Biometrika*, *88*, 551–564.

Ibrahim, J. G., & Molenberghs, G. (2009). Missing data methods in longitudinal studies: A review. *Test*, *18*, 1–43.

Laird, N. M. (1988). Missing data in longitudinal studies. *Statistics in medicine*, *7*, 305–315.

Little, R. J., & Rubin, D. B. (2019). *Statistical analysis with missing data* (Vol. 793). John Wiley & Sons.

Lyu, Z., & Welsh, A. H. (2021a). Asymptotics for EBLUPs: Nested error regression models. *Journal of the American Statistical Association*, *0*, 1–15.

Lyu, Z., & Welsh, A. H. (2021b). Increasing cluster size asymptotics for nested error regression models. *Journal of Statistical Planning and Inference*, *217*, 52–68.

Neuhaus, J. M., McCulloch, C. E., & Boylan, R. (2013). Estimation of covariate effects in generalized linear mixed models with a misspecified distribution of random intercepts and slopes. *Statistics in medicine*, *32*, 2419–2429.

Ning, X., Hui, F. K., & Welsh, A. H. (2024). Asymptotic results for penalised quasi-likelihood estimation in generalised linear mixed models. *Statistica Sinica*. https://doi.org/10.5705/ss.202023.0343

Ployhart, R. E., Bliese, P. D., & Strizver, S. D. (2025). Intensive Longitudinal Models. *Annual Review of Organizational Psychology and Organizational Behavior*, *12*, 343–367.

Pratesi, M. (2016). *Analysis of poverty data by small area estimation*. John Wiley & Sons.

Vonesh, E. F. (1996). A note on the use of laplace's approximation for nonlinear mixed-effects models. *Biometrika*, *83*, 447–452.

Wu, K., & Wu, L. (2007). Generalized linear mixed models with informative dropouts and missing covariates. *Metrika*, *66*, 1–18.

A Additional simulation results

In the presentaton below, we have extracted the $m = 50$ row from Tables 1, 2 and 3 in the main text and appended the $n = 5$ column (results of this are presented in cyan). We have chosen to present the results in such a manner so as to more increasingly draw conclusions around the effect of increasing cluster size from the new smallest value of $n = 5$ to larger values previously investigated e.g., the noticeably and expected and wider interval widths incurred by all methods.

Table 1: **Empirical coverage probabilities of prediction intervals for $\dot{b}_1$, constructed using `glmmTMB`, `lme4`, and the unconditional approach of Ning et al., 2024.**

		Random Intercept					Random Slope				
	m	$n=5$	$n=25$	$n=50$	$n=100$	$n=200$	$n=5$	$n=25$	$n=50$	$n=100$	$n=200$
Unconditonal	50	0.991	0.964	0.954	0.954	0.954	0.986	0.963	0.956	0.947	0.954
glmmTMB	50	0.942	0.932	0.941	0.953	0.950	0.921	0.945	0.946	0.952	0.950
lme4	50	0.916	0.847	0.810	0.719	0.623	0.836	0.783	0.737	0.615	0.499

Table 2: **Empirical average interval lengths of prediction intervals for $\dot{b}_1$, constructed using `glmmTMB`, `lme4`, and the unconditional approach of Ning et al., 2024.**

		Random Intercept					Random Slope				
	m	$n=5$	$n=25$	$n=50$	$n=100$	$n=200$	$n=5$	$n=25$	$n=50$	$n=100$	$n=200$
Unconditional	50	3.466	1.277	0.977	0.783	0.687	3.510	1.082	0.824	0.699	0.633
glmmTMB	50	2.050	1.077	0.880	0.749	0.662	1.531	0.910	0.779	0.682	0.618
lme4	50	1.916	0.901	0.660	0.477	0.336	1.356	0.678	0.513	0.364	0.245

Table 3: **Scaled empirical interval length variance of prediction intervals for $\dot{b}_1$, separately constructed using `glmmTMB` and `lme4`.**

		Random Intercept					Random Slope				
	m	$n=5$	$n=25$	$n=50$	$n=100$	$n=200$	$n=5$	$n=25$	$n=50$	$n=100$	$n=200$
glmmTMB	50	0.108	0.107	0.108	0.112	0.088	0.149	0.087	0.074	0.068	0.049
lme4	50	0.144	0.160	0.188	0.244	0.244	0.101	0.147	0.156	0.180	0.160
